# Structurally Mapping Antibody Repertoires

**DOI:** 10.3389/fimmu.2018.01698

**Published:** 2018-07-23

**Authors:** Konrad Krawczyk, Sebastian Kelm, Aleksandr Kovaltsuk, Jacob D. Galson, Dominic Kelly, Johannes Trück, Cristian Regep, Jinwoo Leem, Wing K. Wong, Jaroslaw Nowak, James Snowden, Michael Wright, Laura Starkie, Anthony Scott-Tucker, Jiye Shi, Charlotte M. Deane

**Affiliations:** ^1^Department of Statistics, Oxford University, Oxford, United Kingdom; ^2^UCB Pharma, Slough, United Kingdom; ^3^Division of Immunology, Children’s Research Center, University Children’s Hospital, Zurich, Switzerland; ^4^Oxford Vaccine Group, University of Oxford, NIHR Oxford Biomedical Research Centre, Oxford, United Kingdom

**Keywords:** antibody specificity, B-cell receptor, next-generation sequencing, structural homology, protein, bioinformatics tools

## Abstract

Every human possesses millions of distinct antibodies. It is now possible to analyze this diversity *via* next-generation sequencing of immunoglobulin genes (Ig-seq). This technique produces large volume sequence snapshots of B-cell receptors that are indicative of the antibody repertoire. In this paper, we enrich these large-scale sequence datasets with structural information. Enriching a sequence with its structural data allows better approximation of many vital features, such as its binding site and specificity. Here, we describe the structural annotation of antibodies pipeline that maps the outputs of large Ig-seq experiments to known antibody structures. We demonstrate the viability of our protocol on five separate Ig-seq datasets covering ca. 35 m unique amino acid sequences from ca. 600 individuals. Despite the great theoretical diversity of antibodies, we find that the majority of sequences coming from such studies can be reliably mapped to an existing structure.

## Introduction

Antibodies are proteins that play a key role in recognizing potentially noxious molecules (antigens) in jawed vertebrates. They are produced by B-cells, where they can be secreted or act as a membrane-bound B-cell receptor. In humans, they are composed of two polypeptide chains, referred to as heavy and light. Each of the chains has a variable region that is ca. 110 amino acids long, composed of the framework region and three hypervariable loops referred to as complementarity determining regions (CDRs). There is a limited set of known human germline framework sequences (~150), but CDRs, which dictate antigen recognition, show more variation (1). It is estimated that a typical human is capable of producing more than 10^10^ distinct antibody molecules (2–6). Thus, in a single individual, there is likely to exist an antibody capable of recognizing an arbitrary antigen, though perhaps not specifically. Such binding malleability of antibodies has long been a subject of intensive academic and industrial research.

Discerning human antibody diversity will help us to understand how our immune system is capable of recognizing such a myriad set of antigens and underpins our ability to exploit them therapeutically (7–10). Next-generation sequencing of immunoglobulin genes (Ig-seq) facilitates this task as it allows us to obtain a snapshot of the B-cell receptor (antibody) repertoire across different individuals and immune states (11–16). The outputs from these Ig-seq experiments have been characterized by their germline biases and sequence analysis methods (2, 10, 14, 17, 18). These studies do not consider the three-dimensional structure of the antibody, but it is this structure that dictates antigen recognition (19, 20). In one study, the authors structurally characterized a small portion of their Ig-seq data (ca. 2,000 structural models from ca. 175,000 sequences) but they did not produce a structural annotation protocol (21). In this paper, we show it is possible to characterize structurally large percentages of the data and describe a pipeline for automating the task.

As described by Kovaltsuk et al. (20), structural information can give both an overall predicted shape and detail of the binding site (CDRs). Predicting the shape of a sequence can offer sufficient information to link it to an antibody with similar shape and defined antigen specificity (22, 23). Enriching Ig-seq datasets with structural information should improve analyses and insights that can be derived from antibody repertoire snapshots.

Here, we describe the structural annotation of antibodies (SAAB) algorithm to bridge the sequence-structure gap in antibody repertoire analysis. This protocol, given a FASTA file with potentially millions of antibody sequences, maps the full sequences, frameworks, and CDRs to the high quality antibody structures currently available in the Protein Data Bank (PDB) (24, 25). We demonstrate the validity of this approach by testing the protocol on five separate Ig-seq datasets encompassing ca 35 m sequences from ca. 600 individuals. For each dataset, we can associate a majority of frameworks and CDR sequences to an existing antibody structure. This finding recapitulates on a large scale both the structural conservation of the framework and the canonical CDR paradigm (26, 27). More generally, however, we demonstrate that it is currently possible to approximate the structures of entire variable region sequences for most of the data. Therefore, despite the theoretically allowed repertoire diversity, currently observed antibody sequence space appears to employ only a conservative set of structural shapes.

## Materials and Methods

### Structural Annotation of Antibodies

Our SAAB algorithm accepts amino acid sequences in FASTA-formatted input. The algorithm first Chothia-numbers the sequences (28) and then maps (if possible) the sequences to known antibody structures in the PDB (25). These structures are identified for entire variable region as well as for frameworks and CDRs separately. Details of the steps of the protocol are given below.

#### Chothia-Number Sequences

Each of the supplied Ig-seq amino acid sequences are Chothia-numbered (28) using ANARCI (29). The numbering provides a consistent frame of reference for antibody sequences. This allows for sequence identity calculation of the entire sequence as well as regions of the antibody separately (frameworks and CDRs).

#### Antibody Structural Reference

The set of antibody structures accompanying our software and used in this analysis was downloaded from the structural antibody database (SAbDab) on 31st October 2017 (25). Only X-ray structures with resolution better than 3.0 Å were used. VHH structures were excluded. If both heavy and light sequences were identical across two or more molecules, only the structure with the best resolution was retained. This procedure resulted in 2,100 antibody structures. Each of the antibody chains is Chothia-numbered using ANARCI. We used these numbered structures to calculate the expected structural difference between two antibodies at a given sequence identity. We describe this structural difference using root mean square deviation (RMSD). RMSD estimates for values below 90% Chothia sequence identity were calculated using the subset of 711 non-redundant structures (less than 90% sequence identity) of the original 2,100 structures. The RMSD estimates above 90% sequence identity in Figure 2 were calculated using the entire set of 2,100 sequences. The RMSD calculation was done using TM-align (30).

#### Chothia-Aligning Full Sequences and Frameworks

Each amino acid sequence from an input Ig-seq dataset is Chothia-aligned to each of the 2,100 antibody structures. The sequence identity is normalized by the length of the query sequence. Chothia positions from the query sequence which cannot be found in the template are treated as mismatches. Sequence identity is calculated for the entire variable region and for the framework region separately. For framework alignments, we employ Chothia definitions of the CDR boundaries. Following this procedure, the PDB codes and chains of best full variable region and framework matches and the corresponding sequence identities are saved for each Ig-seq query sequence.

#### CDR Annotations

The original FREAD algorithm (31, 32) requires a database of fragments from which to select its predictions. In this case, for each CDR a separate database containing only CDRs of that type is used. FREAD also requires as minimal inputs the sequence of the loop to be modeled and the atomic coordinates of the two residues from the framework either side of the loop to be predicted (the anchor residues). Sequence identity of 50% or better between two antibody variable domains gives an anchor RMSD of around 0.4 Å or better. Thus, for a given CDR in a query sequence, we use the best full-sequence match to an existing antibody structure from step 2.1.3 as the anchor reference. From that point, the algorithm runs as described in the original papers [see Ref. (31, 32) for details] but with a length-dependent cutoff for the ESS score. This change was needed as FREAD had originally been benchmarked primarily on loops less than 13 residues long. For loop lengths below 13 the ESS cutoff is maintained at 25, between 13 and 16 the cutoff is set at 40, and for lengths greater than 16 the cutoff becomes 55. These thresholds were established by doing a one versus all cross-validation on a non-redundant set of antibodies in SAbDab at a 20% false positive rate (model has RMSD better than 1.5 Å with respect to the native structure). The anchor coordinates of the reference structure are used to perform a standard loop-template search for the query CDR sequence, using FREAD. If FREAD finds loops with closely matching anchors and an ESS score above the length-dependent cutoff, the best-scoring loop template’s PDB code and chain identifier are saved.

#### Output

For each query sequence, we output (if any) the PDB codes of the best full sequence matches, best framework matches, and best CDR matches. We also output the PDB codes that the largest number of query sequences were mapped to, allowing for large-scale structural characterization of the dataset. If an antibody structure (PDB code) has a bound antigen, these data are also retained to facilitate putative specificity annotation by rationale of sharing similar binding site.

### Data

Four of the datasets (HBP, HBB, MEN, and FLU) were designed to study the immunization effects of vaccines targeting hepatitis B virus (primary and booster vaccination), meningococcus, and influenza virus, respectively. Nucleotide-deposited data for each of the datasets was downloaded from the NCBI website and translated to amino acid sequences. The original dataset sequences were separated by individuals, immunization visits (pre-vaccination or days post-vaccination), and isotype (IgG or IgM). For the purposes of this study, all the sequences from a given dataset were pooled together. These datasets are comprised exclusively of heavy chains. The UCB dataset was designed by UCB Pharma to be a comprehensive snapshot of baseline/naive human antibody diversity. This dataset consists of unpaired heavy and light IgM chains sourced from 494 individuals sampled from various immune organs. Sequences were not separated by individuals. Further details of generation of the datasets can be found in the Section Materials and Methods in Supplementary Material.

### Availability

The SAAB protocol is available as a webserver which performs rapid structural mapping of individual sequences and indicates antigen targets of antibodies with similar binding sites. We also make the code available to perform bulk structural characterizations of large volumes of Ig-seq datasets locally. The amino acid sequences for datasets HPB, HBB, MEN, and FLU are freely available through our site. For the proprietary UCB_H and UCB_L datasets, a sequence-representative dataset, separate frameworks, and CDRs are made available. All the materials are available *via* http://antibodymap.org.

## Results

### The SAAB Algorithm

**Figure 1:**
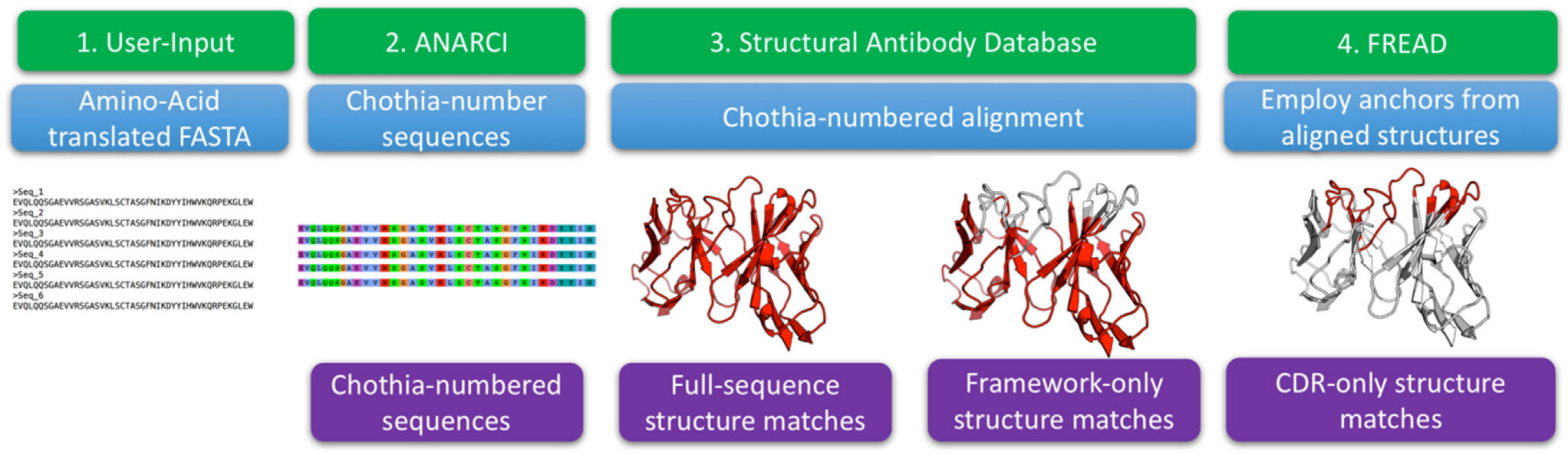
The structural annotation of antibodies algorithm. The input consists of amino acid sequences in FASTA format. These sequences are Chothia-numbered using ANARCI (29). Chothia-numbered sequences are then aligned to known structures of antibodies as defined by the structural antibody database (25). Best templates are identified for the entire variable region as well as for Chothia-delimited framework only. The full variable region templates are employed to define complementarity determining region (CDR) anchoring residues that are used as input to FREAD which determines if we can identify a suitable template for each of the CDRs.

The three-dimensional structures of antibodies define their binding properties and allow a more insightful analysis of these molecules (20). Currently, such structural information is missing from analyses of Ig-seq repertoires (20). In order to structurally enrich outputs of Ig-seq experiments, we have developed an algorithm for the SAAB, see Figure 1. The method is based on previously published ABodyBuilder and FREAD protocols that produce full models of the variable regions and protein loops, respectively (32, 33). These methods were validated by blind prediction of large structural datasets.

The algorithm input consists of FASTA-formatted, amino acid antibody sequences. The input sequences are Chothia-numbered using ANARCI (29). The numbered sequences are Chothia-aligned to 2,100 high quality antibodies with known structure from the SAbDab (25). For each sequence from an Ig-seq dataset, the alignment identifies the best structural templates for the full variable region and framework separately. FREAD then identifies, if any, the most suitable template structures for the CDRs (31, 32).

To demonstrate the efficacy of our protocol, we applied SAAB to five diverse Ig-seq datasets: one comprehensive dataset from ca. 500 people as well as four immunization datasets, each containing ~5 m unique amino acid sequences (Table 1).

**Table 1:** Sequence datasets.

Dataset name	Non-redundant sequences (H = heavy chain, L = light chain)	Individuals	Description
UCB_H	H: 4,925,532	494 (pooled)	Proprietary, non-immunized comprehensive diversity library
UCB_L	L: 8,380,540		
HBP	H: 7,685,149	15	Hep B Primary vaccination (34)
HBB	H: 4,718,120	10	Hep B Booster (12)
MEN	H: 6,036,457	10	Meningococcal vaccination (35)
FLU	H: 3,409,916	58	Influenza vaccination (36)

*We have employed one dataset of baseline antibody human diversity (UCB_L and UCB_H) and four immunized datasets (HBP, HBB, MEN, and FLU). In total, the datasets comprised ca. 600 individuals and ca. 36 m sequences*.

### The Majority of Full Variable Regions and Frameworks From Ig-seq Outputs Can be Reliably Matched to Available Structures

We quantified the proportion of sequences in each of our Ig-seq datasets for which it is possible to find a suitable structural template. These closest structural matches tell us how well we can model each of the V-regions. Given the size of our Ig-seq datasets, the results indicate how well currently available structures potentially represent the general shape adopted by immunoglobulins.

**Figure 2:**
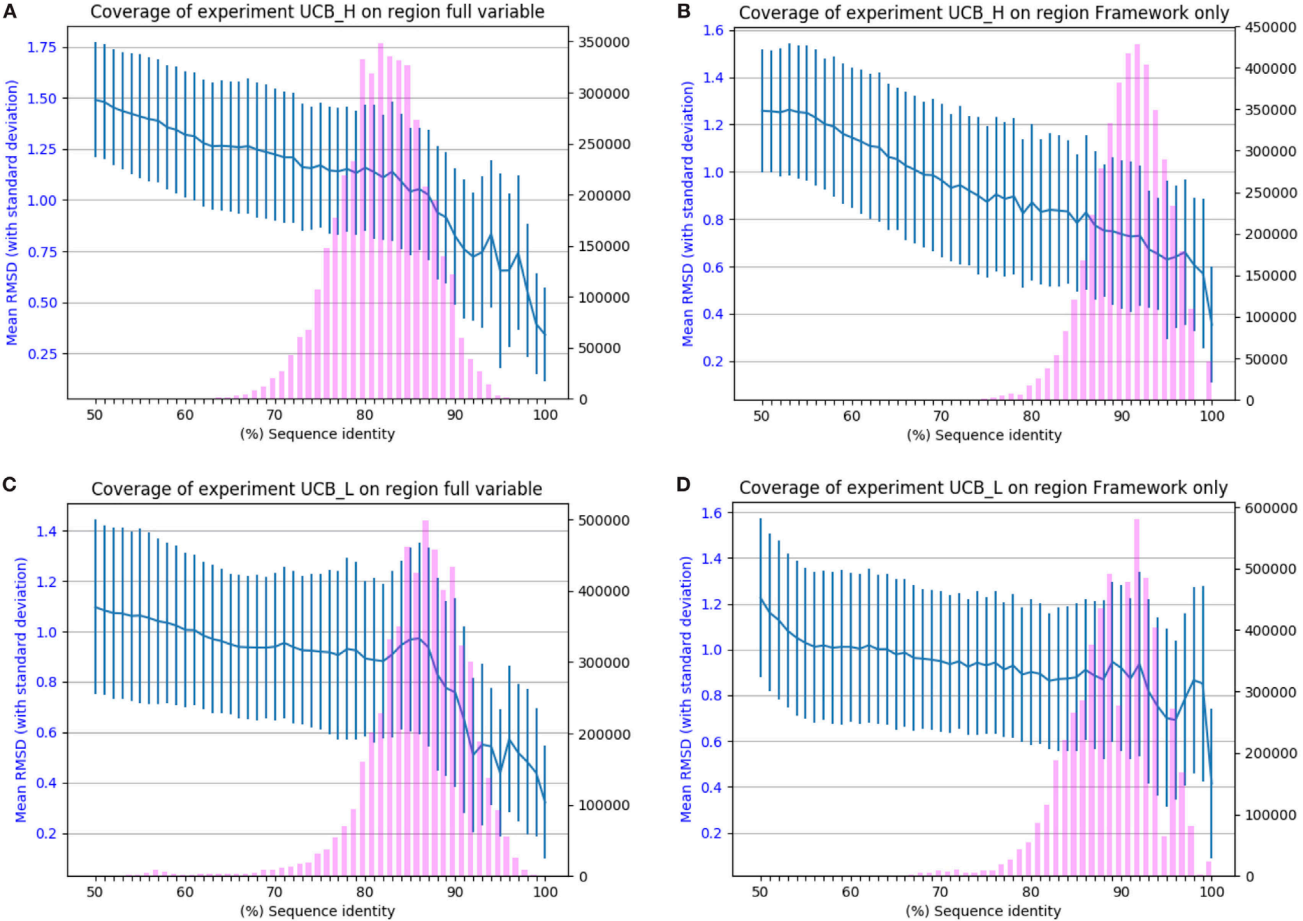
Chothia-aligning the 13.5 m unique baseline antibody variable sequences in datasets UCB_H and UCB_L to antibodies with known structures. **(A)** Full variable region sequence of the heavy chain. **(B)** Framework of the heavy chain. **(C)** Full variable region of the light chain. **(D)** Framework of the light chain. The pink bars indicate the number of sequences (right-hand *y*-axis) whose highest sequence identity structure match has the sequence identity given on the *x*-axis. The blue line (left-hand *y*-axis) indicates the expected root mean square deviation (RMSD) of a model built using a sequence identity match of that quality (with vertical SD error bars). For example, 80% sequence identity for the framework of the heavy chain translates to a 0.8 Å expected model RMSD.

The results of applying SAAB to the UCB_L and UCB_H datasets are shown in Figure 2. The blue lines indicate the RMSD that can be expected if a structural model was produced from a template with the sequence identity given on the *x*-axis [structures of 1.5 A° RMSD or better can be considered close to identical (1)]. The bars indicate the numbers of sequences in the Ig-seq dataset whose best structural template identified is of the corresponding sequence identity. These results suggest that there are few if any antibody framework structures unaccounted for as 97% of heavy chain and 94% of light chain frameworks in our dataset align to a PDB structure with over 80% sequence identity (thus expected model RMSD of 0.9 A° or better). The coverage is also similar for the entire variable region, suggesting that for the vast majority of sequences in the naïve human antibody repertoire sampled by datasets UCB_H and UCB_L we would be able to produce a structural model closely resembling the native structure. The blue line shows that even with only 50% sequence identity an antibody framework would be predicted accurately. This shows the close structural homology of the framework sequences across V gene families (see Section 1 in Supplementary Material).

The other four datasets show a similar pattern (see Section 2 in Supplementary Material), which suggests that there are very few, if any “blind spots” in our knowledge of antibody domain structure. The antibody repertoire, as represented by the five datasets, appears to consist of a reasonably conservative set of scaffolds.

Therefore, using only 2,100 high quality antibody structures, it is possible to group Ig-seq space into general structural groups. Such broad-brush characterization can offer valuable information for structurally informed comparisons of sequences, but fails to address antibody specificity. In order to be able to draw conclusions about antibody specificity, it is necessary to map structurally the antibody binding site as defined by the CDRs.

### Majority of CDR Sequences From Ig-seq Datasets Can be Matched to an Existing Structural Template

We calculated the number of CDR sequences in our Ig-seq datasets for which we could find structural templates. The existence of a suitable structural template indicates that it is possible to produce a structural model of the loop. This, in turn, allows an approximation of the antibody binding site shape providing insight into its specificity.

For each Ig-seq dataset, we extracted the Chothia-defined CDR loops and grouped them by CDR type (H1, L2, etc.). Loops which were shorter than three amino acids long were discarded. We created redundant and non-redundant sets for each loop (see Table 2). We find that large proportions of the redundant non-H3 CDRs can be found directly in the PDB (Table 2). For instance, of the 4,718,716 H1 redundant loops in the UCB dataset, 2,238,760 (47%) are identical in sequence to a known H1 CDR antibody structure. By contrast, of the 110,495 non-redundant H1 loops in the UCB dataset, only 156 have identical sequence matches in the PDB. Non-H3 CDRs are known to adopt a limited number of canonical shapes so the high coverage of the redundant sequences is expected (27). The large discrepancy between the number of direct PDB matches between redundant and non-redundant datasets may indicate the existence of common loop sequences which thus have higher probability of having being crystallized at some point. We have listed all H1 and H2 loops for which we found a direct PDB match in Section 3 in Supplementary Material. These data show that these CDRs are shared between the five studies. Many of these CDRs are germline. To obtain a more comprehensive structural interpretation of the Ig-seq CDRs for which we do not find direct matches in the PDB, we predicted the loop conformation from sequence.

**Table 2:** Structural mapping of the complementarity determining regions (CDRs) in the UCB_H and UCB_L datasets.

Dataset and CDR subset	Total: redundant (non-redundant)	In the Protein Data Bank (PDB): redundant (non-redundant)	Can model: redundant (non-redundant)	Cannot model: redundant (non-redundant)
UCB_H				
H1	4,718,716 (110,495)	2,238,760 (159)	2,479,674 (110,245)	282 (91)
H2	4,718,717 (159,222)	1,568,190 (305)	3,150,527 (158,917)	0 (0)
H3	4,714,545 (1,623,070)	61 (25)	3,614,289 (1,088,900)	1,100,195 (534,145)

**UCB_L**				
L1	8,127,157 (1,020,446)	889,922 (135)	7,206,821 (1,005,664)	30,414 (14,647)
L2	8,127,157 (159,646)	2,942,147 (189)	5,137,392 (151,627)	47,618 (7,830)
L3	8,120,282 (1,080,668)	135,548 (130)	7,876,402 (1,060,293)	109,332 (20,245)

*Chothia-defined CDRs in each dataset were extracted from the full variable region. CDRs, which had length less than three, were discarded. The redundant datasets were constrained to unique sequences only, which we denote as “non-redundant,” and the resulting numbers of loops are given in the “Total” column. “In the PDB” indicates number of loops we could find direct sequences matches for in the PDB. Of the loops, which were not found directly in the PDB, the “Can model” column indicates the number of loops FREAD found suitable templates for. The “Cannot model” column shows the numbers of loops which were not in the PDB and FREAD could not find templates for. In each case, the numbers of redundant loops are given without parentheses whereas non-redundant loops are given in parentheses*.

The structural templates for each CDR are identified using an adapted version of the loop-modeling algorithm FREAD (32) which was benchmarked for this particular study (see Materials and Methods). Given a query sequence, the algorithm determines if there is a fragment or loop in a pre-compiled structural database (in this case antibodies in the PDB) that would serve as a reliable modeling template (<1.5 Å RMSD). FREAD gives no prediction if it fails to find a close match. This allows us to produce an estimate for the percentage of CDR sequences we could reliably predict the structure of.

Using FREAD, we found that we could produce a reliable model for the majority of the CDR sequences in the UCB dataset (Table 2). Well over 50% of the unique sequences of non-H3 CDRs can be accurately structurally predicted. This appears to recapitulate on a large scale the canonical shape phenomenon of non-H3 CDRs. In the case of H3, even though there were only a handful of direct PDB matches to UCB_H H3 sequences, according to FREAD estimates, we would be able to produce structural models for 65% of all non-redundant and 75% of redundant H3 loops. This is a surprisingly high coverage given its theoretical sequence diversity and lack of canonical rules.

Similar results are seen for CDRs in the other four datasets (see Section 1 in Supplementary Material). Given the theoretically allowed diversity of antibody binding sites, these results suggest that the immune system is using only a limited set of CDR sequences and an even more limited set of CDR backbone shapes to generate diversity.

## Conclusion

The diversity of the human antibody repertoire renders its characterization a challenging task. Newly available Ig-seq protocols allow us to take snapshots of this diversity across different individuals, isotypes, and immune states. Current Ig-seq analysis pipelines rely on sequence-only methods to gain insight into antibody mechanics. However, it is the three-dimensional structure of the antibody that defines the specific physicochemical configuration which modulates the molecule’s specificity and affinity. Enriching Ig-seq datasets with structural information would greatly help in drawing biological or therapeutic conclusions about sequencing data (20).

In order to bridge the gap between high-throughput antibody sequencing and three-dimensional structure we developed SAAB. Our algorithm identifies the most suitable (if any) structural templates for the entire variable region as well as the framework and CDR regions alone. We tested the method on five datasets containing over 36 m sequences and show that the 2,100 high quality antibody structures in the public domain are sufficient to structurally annotate the majority of sequences.

The ability to identify structural templates for the majority of frameworks is expected since these are drawn from a limited set of known germlines. Similarly, non-H3 CDRs are known to adopt a set of canonical shapes. Furthermore, there appears to be a large proportion of non-H3 CDR shapes that are highly re-used in the datasets. Considering the diversity and volume of the datasets involved it may be the case that the majority of the human immune repertoire is composed of a limited, perhaps strategically preferred, set of sequences and an even more conservative set of backbone shapes.

**Figure 3:**
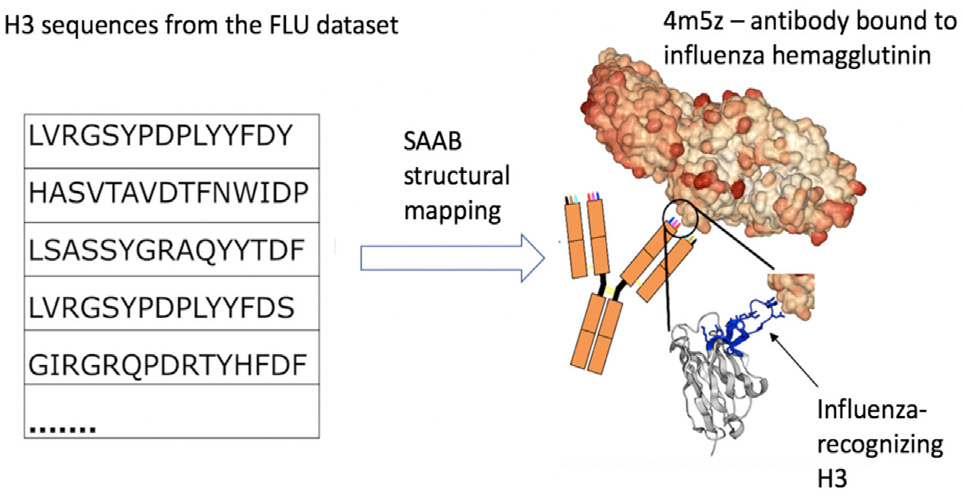
Example of how structural mapping provides clues to antibody specificity. Structural annotation of antibodies (SAAB) outputs the Protein Data Bank (PDB) codes used to map frameworks, full variable sequence, and each of the complementarity determining regions for a sequence. The PDB codes are also mapped to the antigens recognized by the antibody structures (as stored in structural antibody database). If sequences match to similar PDB structures this could be indicative of similar binding sites and thus specificity. As an example, we examined the top 10 PDBs that were used to map H3 in the FLU dataset. A total of more than 7k H3 sequences were mapped to 4m5z, a complex of an antibody with influenza hemagglutinin (this is not among the top 10 H3-mapped PDBs in our other datasets). We show several sequence-diverse H3 loops on the left, which are unlikely to be grouped together by sequence-only methods. However, SAAB identifies that they are all likely to share a similar structure to the H3 loop of 4m5z (right, in blue) and, therefore, perhaps similar specificity.

Binding shape biases in antibody repertoires can offer insight into the strategies of the immune system for tackling arbitrary antigens. For instance, sequence similarities can be indicative of shared antigen specificity (16, 37). However, the particular three-dimensional configuration of the CDRs can help in identifying the physichochemical properties of the paratope, providing potentially clearer information on specificity (21). It was previously shown on a smaller scale that certain CDR length or canonical class combinations can be associated with different types of antigens (38, 39). Thus, sharing of a similar structural template could also be an indication of similar specificity (Figure 3). However, this is only the first step in identifying potential antigen-specific antibodies within the totality of the repertoire. Such candidates would have to be carefully studied with respect to the physicochemical properties of their paratopes, as such non-covalent interactions are known to ultimately dictate antibody-antigen recognition (39, 40). Special focus should be placed on electrostatic interactions that are suggested to be of particular importance in antibody–antigen complexes (41, 42). Annotating sequences with such physicochemical information can only be done reliably given the structure of the antibody (43). Such structural annotations will become more pertinent and accurate as more paired Ig-seq datasets are released as these data allow the entire Fv to be modeled rather than separate heavy and light chains (21). Such disease-specific annotations could be employed in immunodiagnostics to find antibody-markers of known diseases. Therefore, employing structural information provides novel ways to study the diversity of the immune system.

Up until now, structural mapping of entire repertoires was hampered by the computational cost associated with variable region modeling. Nevertheless, just identifying templates without producing a detailed model is in most cases more feasible than producing an ideal structural model and is already representative of shape (1, 21). For instance, rapidly identifying structurally similar groups by shared templates or structural similarity among templates in immunized Ig-seq datasets could be indicative of antigenic specificity. Employing structural annotations in such a fashion could allow for the development of computationally rapid predictive methodology for selecting antibody sequences to experimentally test for binding to an antigen. The inability to better identify antigen-specific sequences from sequence alone has been long proposed as a roadblock to better vaccine development (16).

Furthermore, it has been shown previously that certain antigen-specific sequences can be found across different individuals (44, 45). Such antibodies were proposed to constitute the so-called “public repertoire” indicating sequence convergences within the population to combat certain groups of antigens (45, 46). SAAB annotations will offer an opportunity for in-depth characterization of structural commonalities and divergences in the inter-individual repertoires (46). For instance, comparing the overlap of subsets of structural annotations between individuals of healthy and diseased states could shed light on preferred groups of antibodies used for antigen recognition. The large sequence redundancy we observe here offers further evidence that there are antibodies that might be preferentially used by an organism to carry out immune responses. The high level of structural coverage we observe here suggests that repertoires in general and the public repertoire in particular is structurally conservative, perhaps indicating immune preferences beyond germline genes. Therefore, characterization of antibody repertoires in terms of sequence and structural features will help us to understand the strategic choices made by organisms which can, in turn, improve our ability for therapeutic design of these molecules. SAAB allows researchers to enrich Ig-seq datasets with structural information empowering them to draw greater biologically and therapeutically relevant information from their data.

## Author Contributions

KK, SK, JaS, MW, JiS, and CD conceived the experiments. JaS, LS, MW, AS-T, JG, DK, and JT contributed data. KK, SK, JaS, JN, CR, AK, WW, and JL analyzed the data. KK and CD wrote the manuscript. All authors reviewed the manuscript.

## Conflict of Interest Statement

SK, JS, LS, MW, AS-T, and JS were employed by company UCB Pharma. All other authors declare no competing interests.
