# Supplementary material for: Structurally Mapping Antibody Repertoires
Source: Front Immunol. 2018 Jul 23;9:1698. doi: 10.3389/fimmu.2018.01698 (PMC6064724; doi:10.3389/fimmu.2018.01698)
Supplement: Supplementary file 1 [file data_sheet_1.PDF]

Supplementary Information

Structurally Mapping Antibody Next Generation Sequencing Repertoires.

Authors: Krawczyk K.[1], Kelm S.[2], Kovaltsuk A., Galson J, Kelly D, Trueck J, Regep C.[1], Leem, J.[1], Wong W.K. [1], Nowak J.[1], Snowden J.[2], Wright M.[2], Starkie L.[2], Scott-Tucker A.[2], Shi J.[2], Deane C.M.[1]*

[1] Department of Statistics, Oxford University, Oxford, UK.

[2] UCB Pharma, Slough, UK.

[3] Oxford Vaccine Group, University of Oxford and the NIHR Oxford Biomedical Research Centre, Oxford, UK.

[4] Paediatric Immunology, University Children's Hospital, Zurich, Switzerland.

Section 1. Structural and sequence variability in the germline.

In order to illustrate the sequence variability that can be expected across germline genes we plotted the sequence differences across all possible combination of functional V/J genes in Supplementary Figure 1 (with the caveat of not including the D segment in the IGH alignments). To illustrate the structural conservation across different IMGT gene families we plotted Supplementary Figure 2.

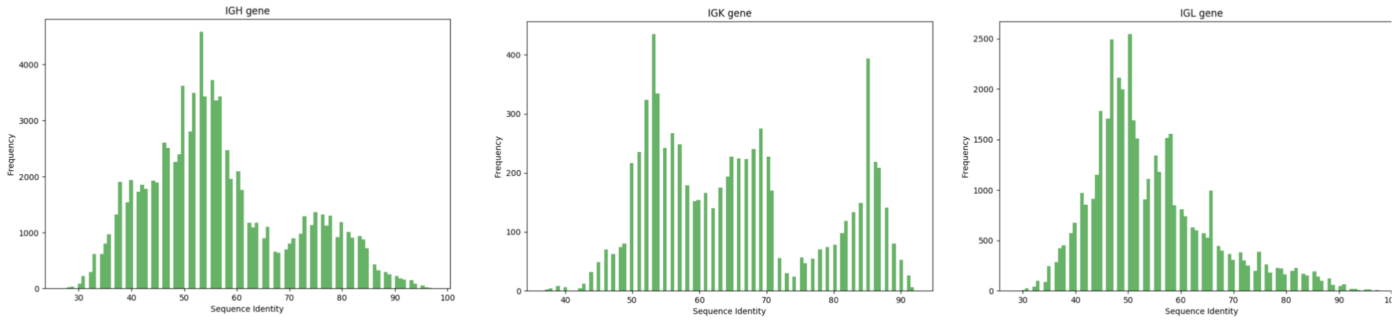

A B C

Supplementary Figure 1. Sequence differences and similarities among germline sequences. We extracted the amino acid sequences for each of IGHV, IGKV, IGLV, JH, JK and JL genes. We created all possible combination of the V and J genes and numbered them using the Chothia scheme. We calculated the sequence identity for each pair where the V and J genes were non-identical. We plot the histogram of the resulting sequence identities for each of IGH (A), IGK (B) and IGL (C).

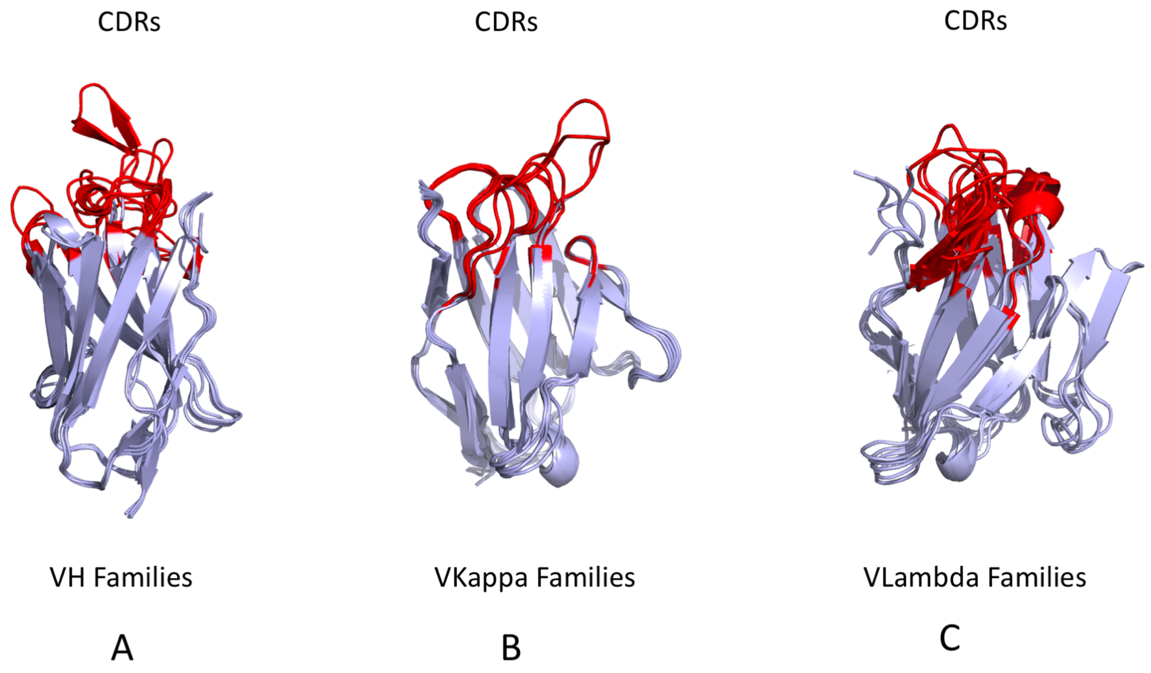

Supplementary Figure 2. Structural uniformity of antibody frameworks. We structurally aligned representatives for each member of the A) IGHV (1vge, 3mod, 1bj1, 1mhp, 3h0t. 3mlr, 3go1), B) IGKV (1efq, 4fz8, 1n0x, 3qcu) and C) IGLV (4bjl, 3t2n, 5hi3, 4kte, 3fl5, 4lsu) genes. Frameworks are shown in blue, CDRs in red. Despite sequence differences between the underlying gene families, the structure is conserved.

Section 2. Structural mapping of MEN, HBB, HBP and FLU datasets.

We have performed structural mapping of four additional datasets, FLU, MEN, HBB and HBP as given in Table 1 in the main manuscript.

HBP Dataset

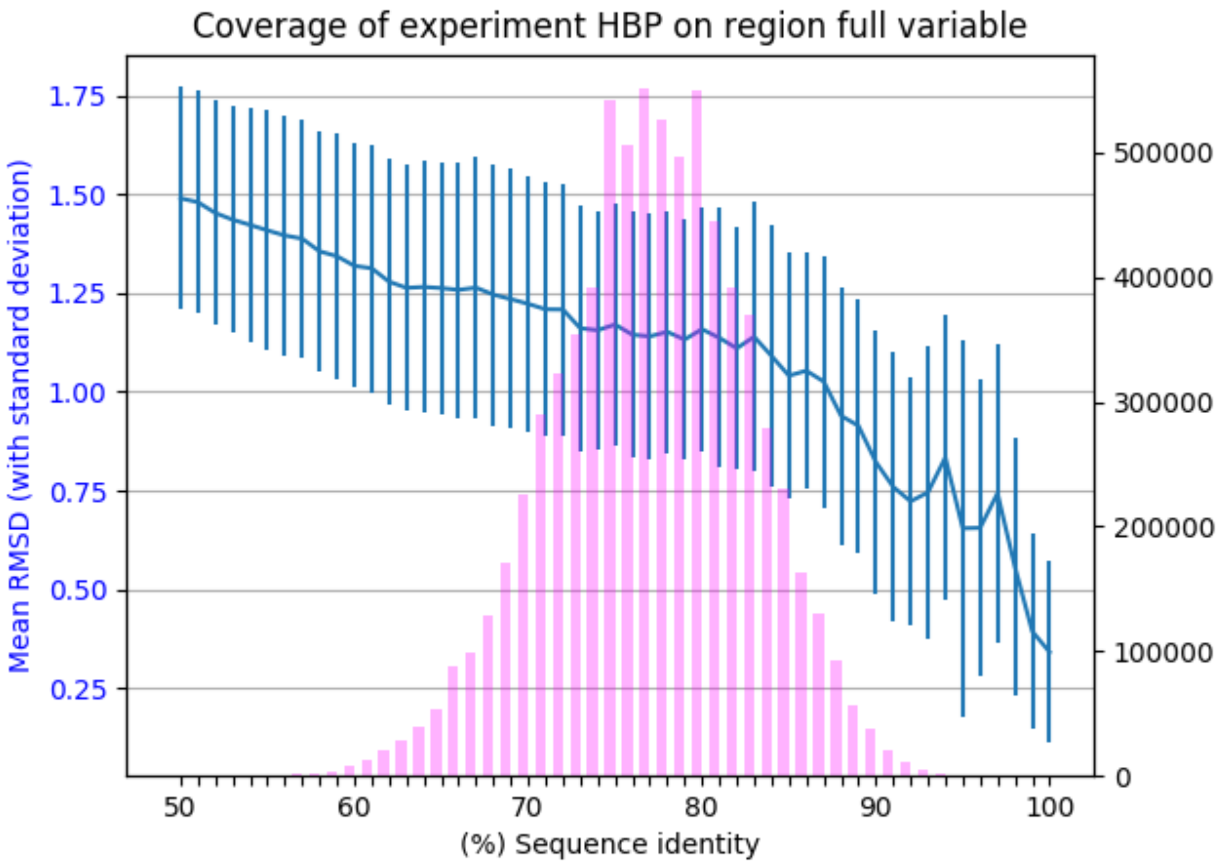

Supplementary Figure 3. Structural coverage of full variable sequences in HBP dataset. The pink bars indicate the number of sequences (right-hand y-axis) whose highest sequence identity structure match has the sequence identity given on the x-axis. The blue line (left-hand y-axis) indicates the expected Root Mean Square Deviation (RMSD) of a model built using a sequence identity match of that quality (with vertical standard deviation error bars).

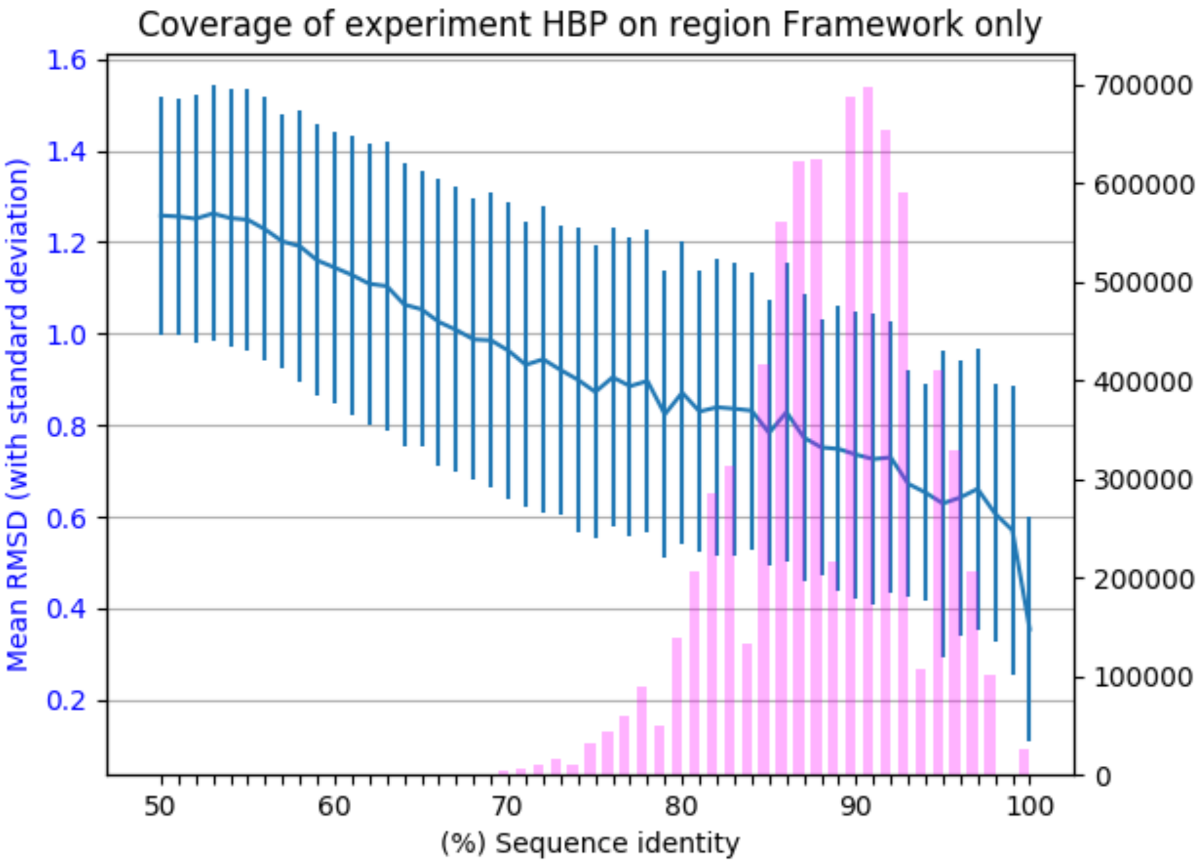

Supplementary Figure 4. Structural coverage of framework sequences in HBP dataset. The pink bars indicate the number of sequences (right-hand y-axis) whose highest sequence identity structure match has the sequen**ce** identity given on the x-axis. The blue line (left-hand y-axis) indicates the expected Root Mean Square Deviation (RMSD) of a model built using a sequence identity match of that quality (with vertical standard deviation error bars).

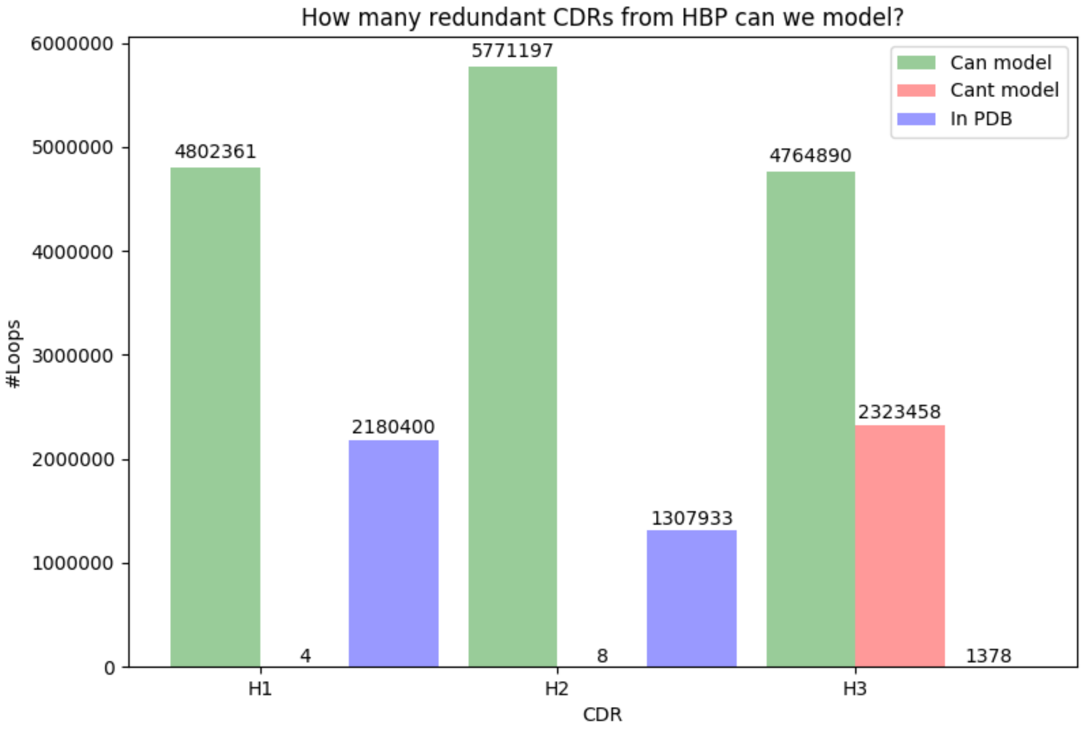

Supplementary Figure 5. Structural coverage of redundant CDRs in HBP. Blue bars indicate CDRs we found direct sequence matches in the PDB for. If no direct PDB match was found but we could produce a structural model, we indicate using green bars. The loops for which we could not find direct matches in the PDB and we could model are indicate by red bars.

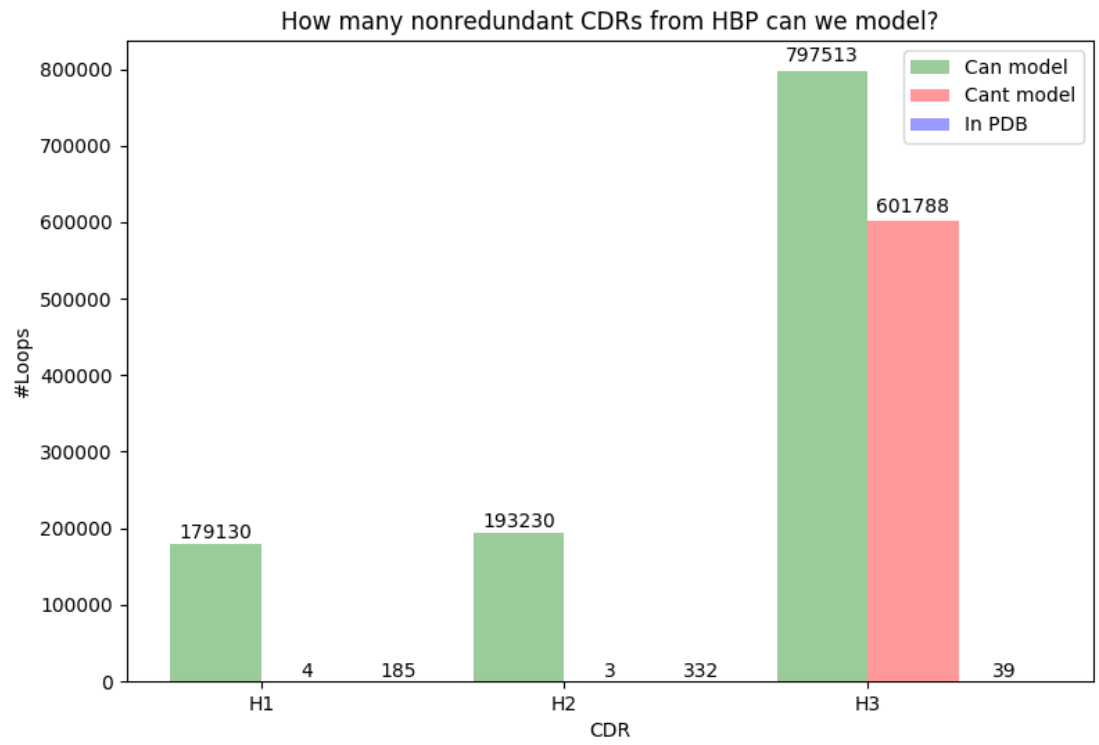

Supplementary Figure 6. Structural coverage of nonredundant CDRs in HBP. Blue bars indicate CDRs we found direct sequence matches in the PDB for. If no direct PDB match was found but we could produce a structural model, we indicate using green bars. The loops for which we could not find direct matches in the PDB and we could model are indicate by red bars.

HBB Dataset

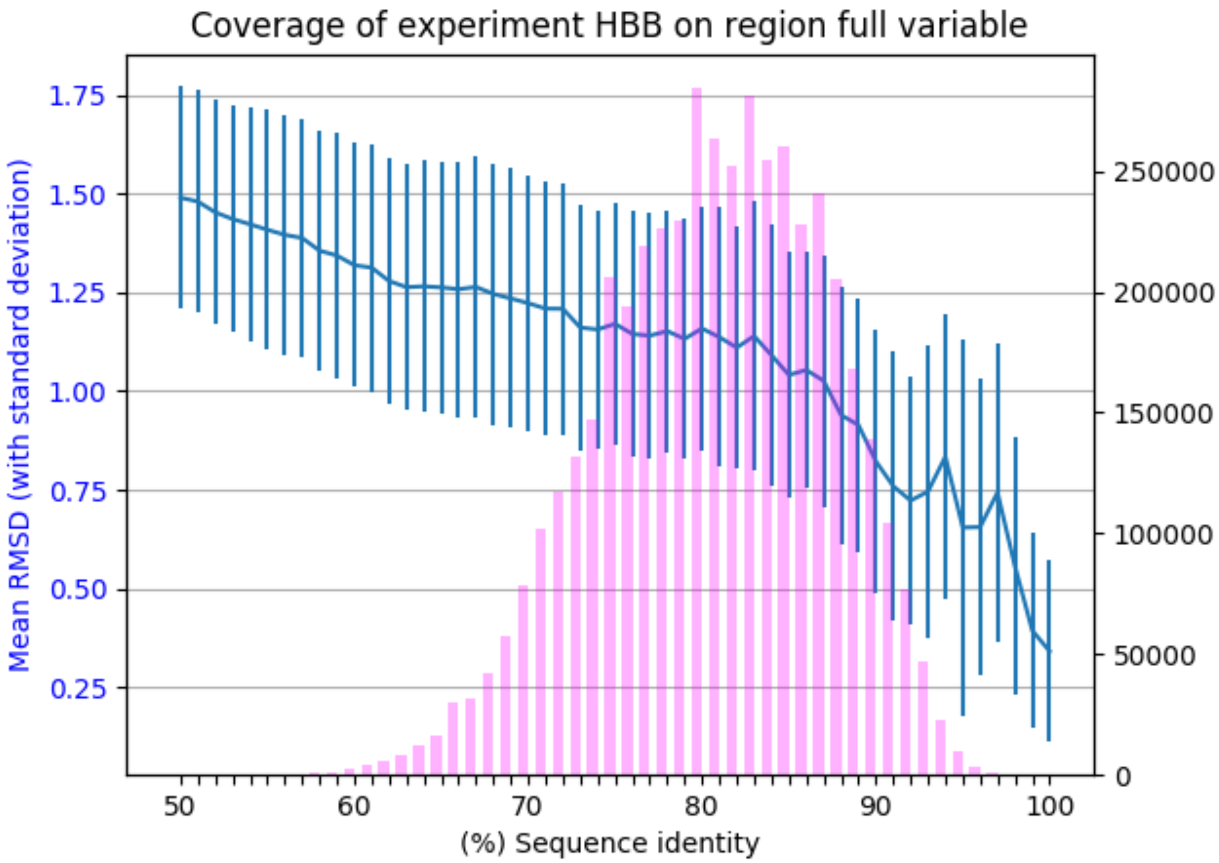

Supplementary Figure 7. Structural coverage of full variable sequences in HBB dataset. The pink bars indicate the number of sequences (right-hand y-axis) whose highest sequence identity structure match has the sequen**ce** identity given on the x-axis. The blue line (left-hand y-axis) indicates the expected Root Mean Square Deviation (RMSD) of a model built using a sequence identity match of that quality (with vertical standard deviation error bars).

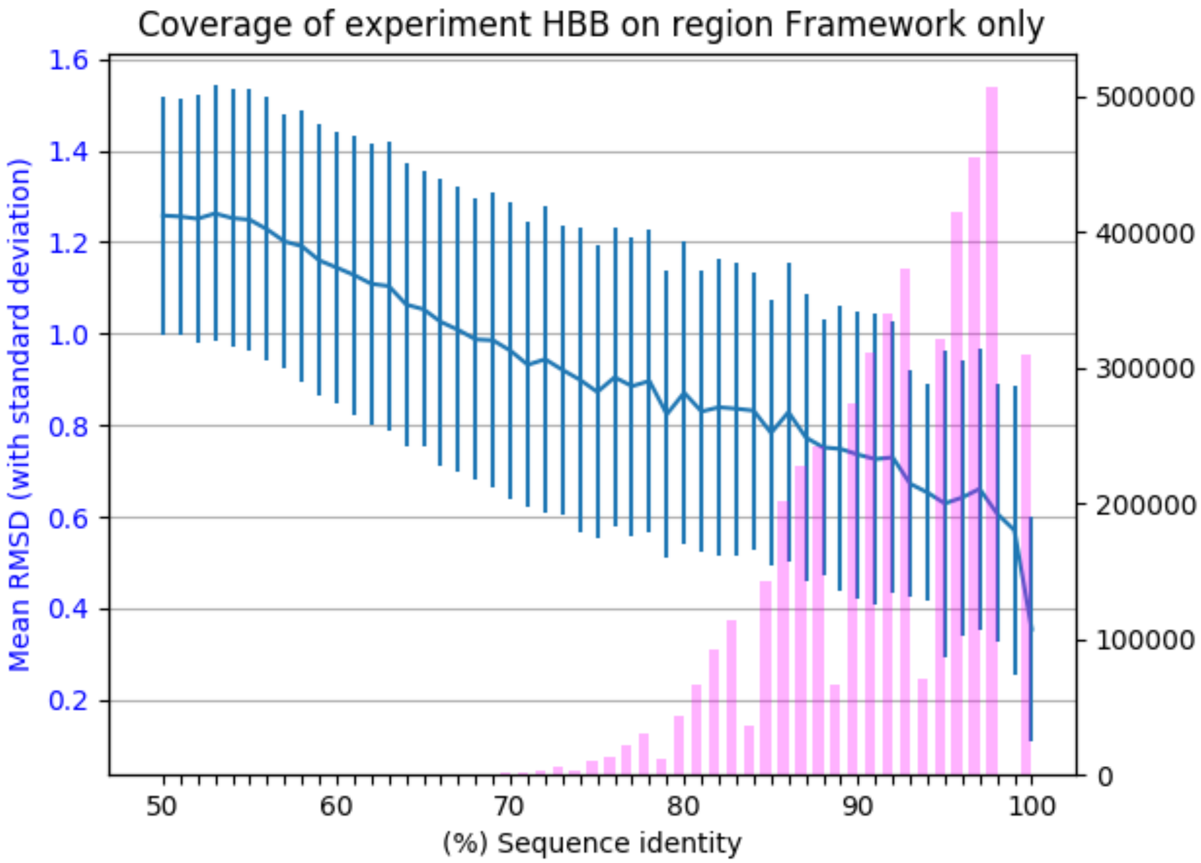

Supplementary Figure 8. Structural coverage of framework sequences in HBB dataset. The pink bars indicate the number of sequences (right-hand y-axis) whose highest sequence identity structure match has the sequen**ce** identity given on the x-axis. The blue line (left-hand y-axis) indicates the expected Root Mean Square Deviation (RMSD) of a model built using a sequence identity match of that quality (with vertical standard deviation error bars).

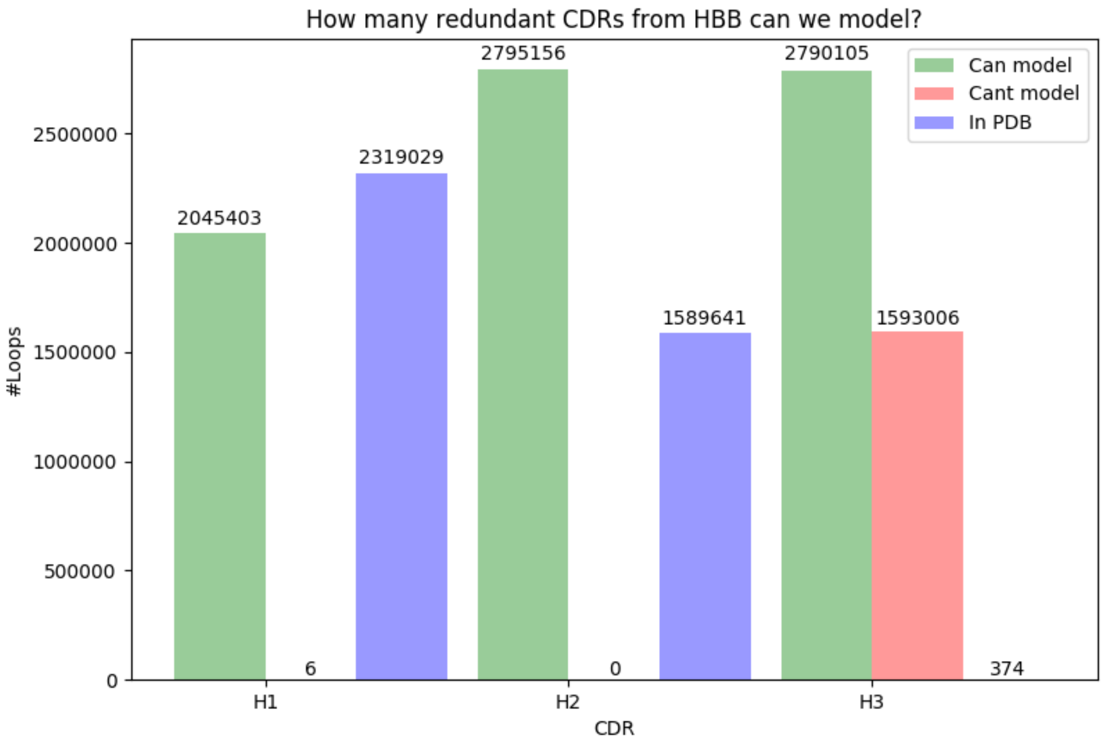

Supplementary Figure 9. Structural coverage of redundant CDRs in HBB. Blue bars indicate CDRs we found direct sequence matches in the PDB for. If no direct PDB match was found but we could produce a structural model, we indicate using green bars. The loops for which we could not find direct matches in the PDB and we could model are indicate by red bars.

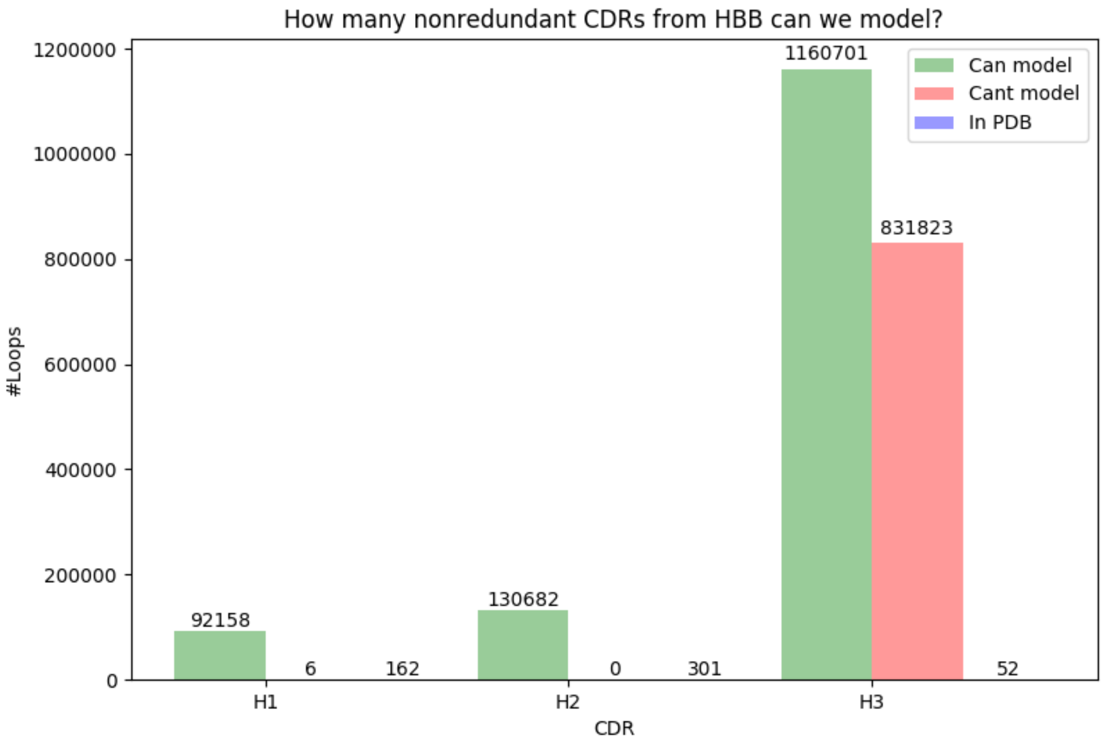

Supplementary Figure 10. Structural coverage of nonredundant CDRs in HBB. Blue bars indicate CDRs we found direct sequence matches in the PDB for. If no direct PDB match was found but we could produce a structural model, we indicate using green bars. The loops for which we could not find direct matches in the PDB and we could model are indicate by red bars.

MEN Dataset

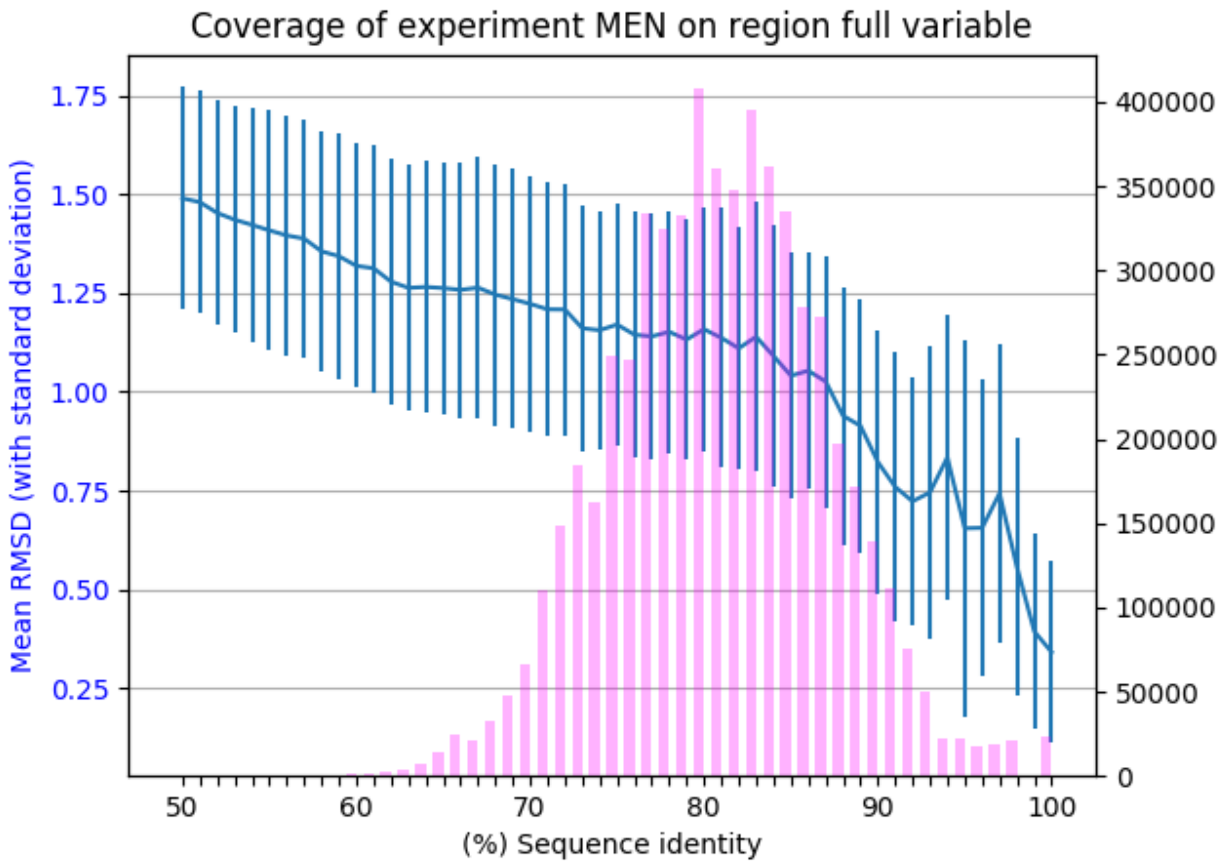

Supplementary Figure 11. Structural coverage of full variable sequences in MEN dataset. The pink bars indicate the number of sequences (right-hand y-axis) whose highest sequence identity structure match has the sequen**ce** identity given on the x-axis. The blue line (left-hand y-axis) indicates the expected Root Mean Square Deviation (RMSD) of a model built using a sequence identity match of that quality (with vertical standard deviation error bars).

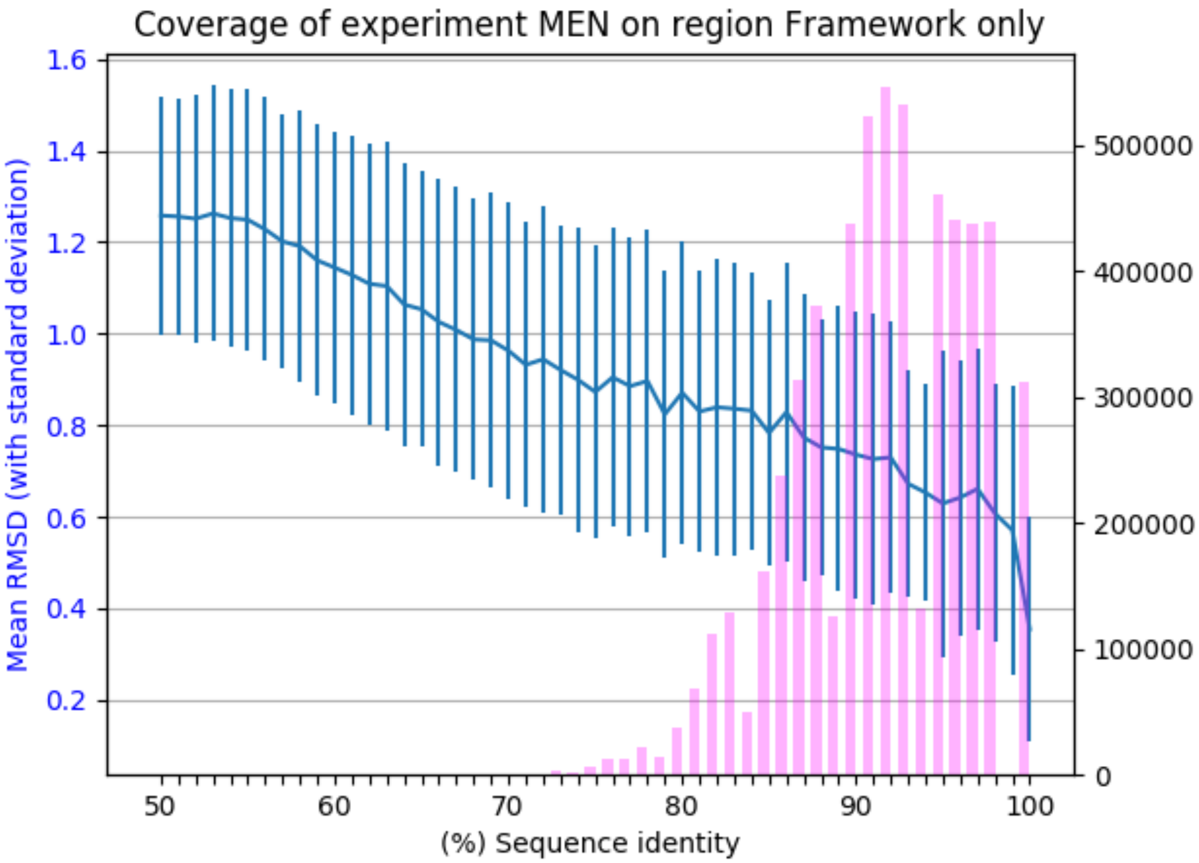

Supplementary Figure 12. Structural coverage of framework sequences in MEN dataset. The pink bars indicate the number of sequences (right-hand y-axis) whose highest sequence identity structure match has the sequen**ce** identity given on the x-axis. The blue line (left-hand y-axis) indicates the expected Root Mean Square Deviation (RMSD) of a model built using a sequence identity match of that quality (with vertical standard deviation error bars).

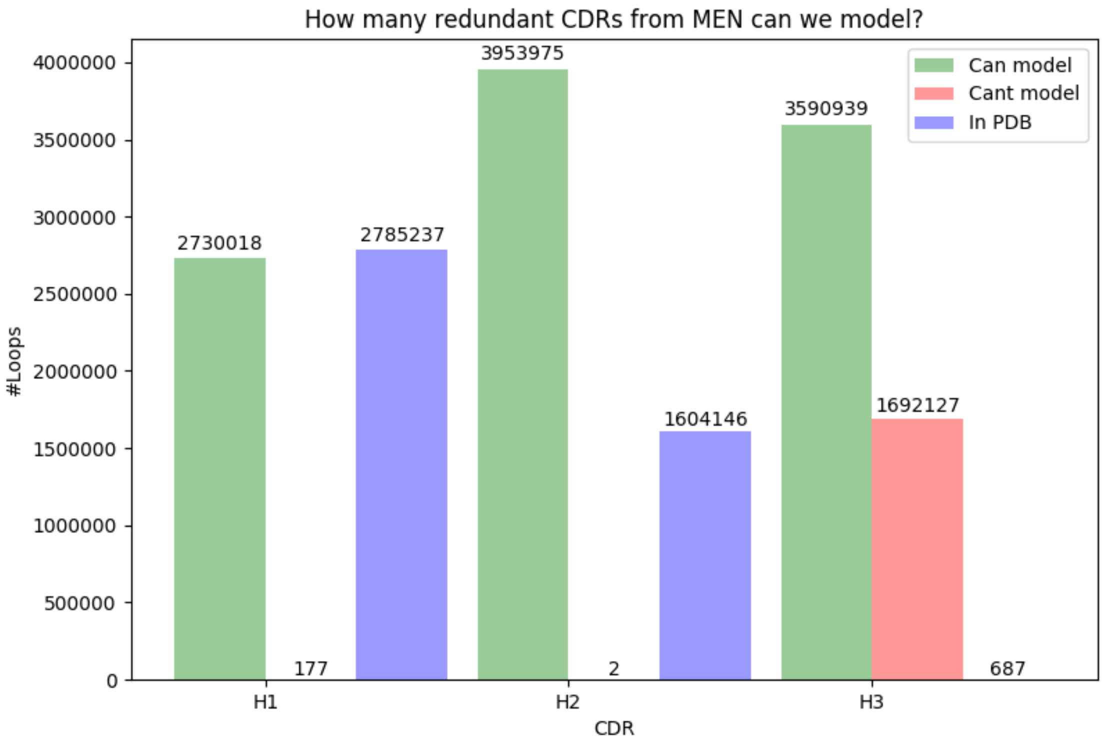

Supplementary Figure 13. Structural coverage of redundant CDRs in MEN. Blue bars indicate CDRs we found direct sequence matches in the PDB for. If no direct PDB match was found but we could produce a structural model, we indicate using green bars. The loops for which we could not find direct matches in the PDB and we could model are indicate by red bars.

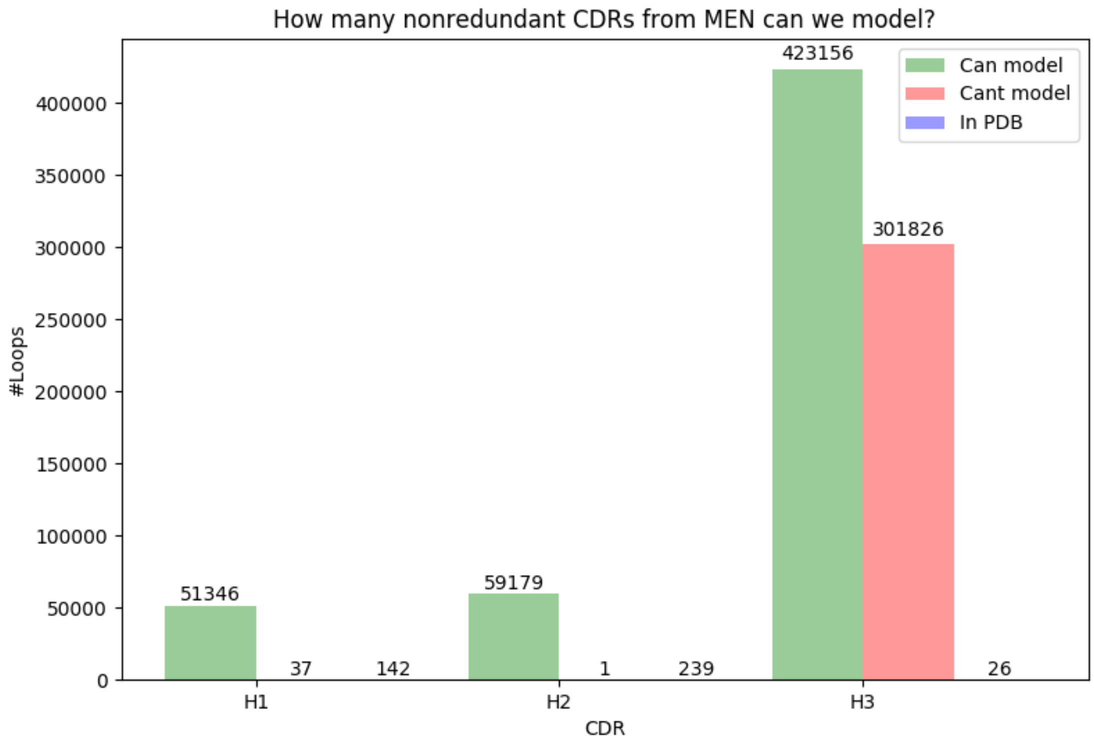

Supplementary Figure 14. Structural coverage of nonredundant CDRs in MEN. Blue bars indicate CDRs we found direct sequence matches in the PDB for. If no direct PDB match was found but we could produce a structural model, we indicate using green bars. The loops for which we could not find direct matches in the PDB and we could model are indicate by red bars.

FLU Dataset

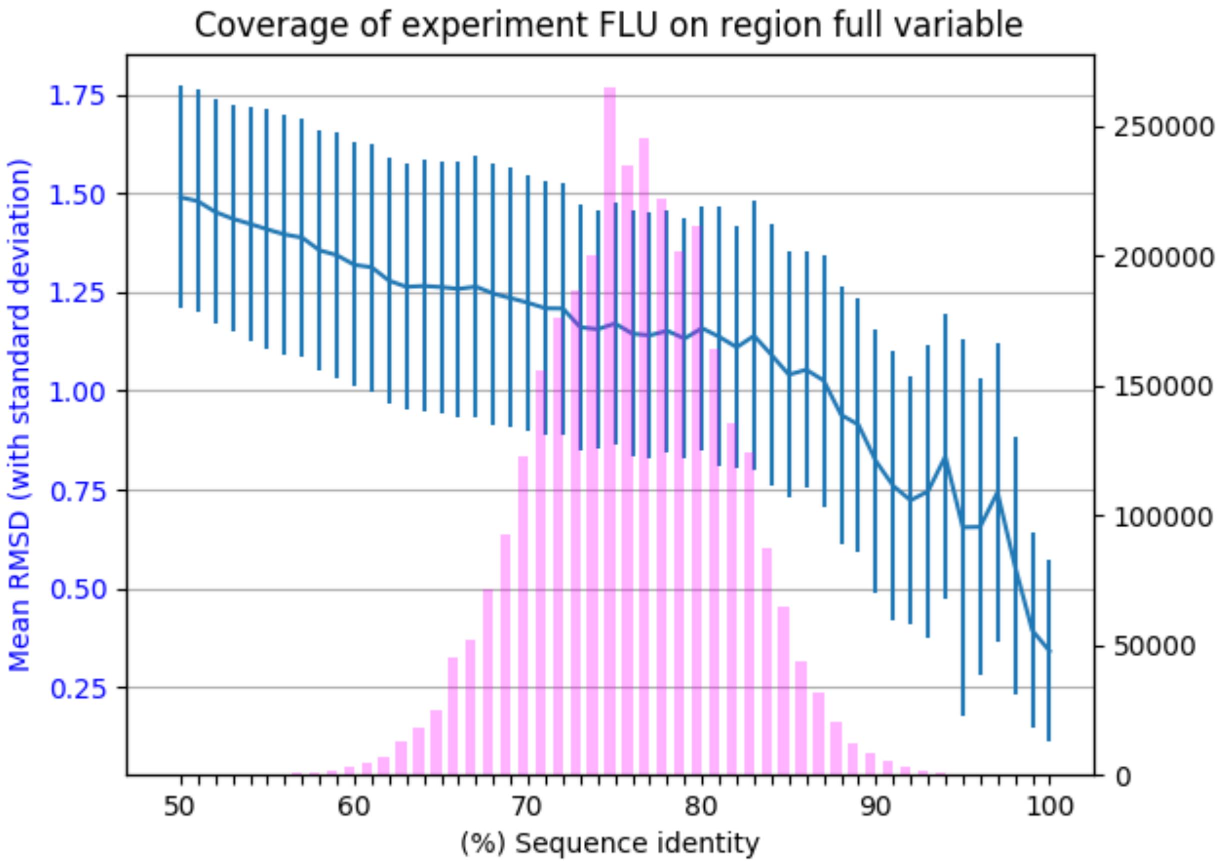

Supplementary Figure 15. Structural coverage of full variable sequences in FLU dataset. The pink bars indicate the number of sequences (right-hand y-axis) whose highest sequence identity structure match has the sequence identity given on the x-axis. The blue line (left-hand y-axis) indicates the expected Root Mean Square Deviation (RMSD) of a model built using a sequence identity match of that quality (with vertical standard deviation error bars).

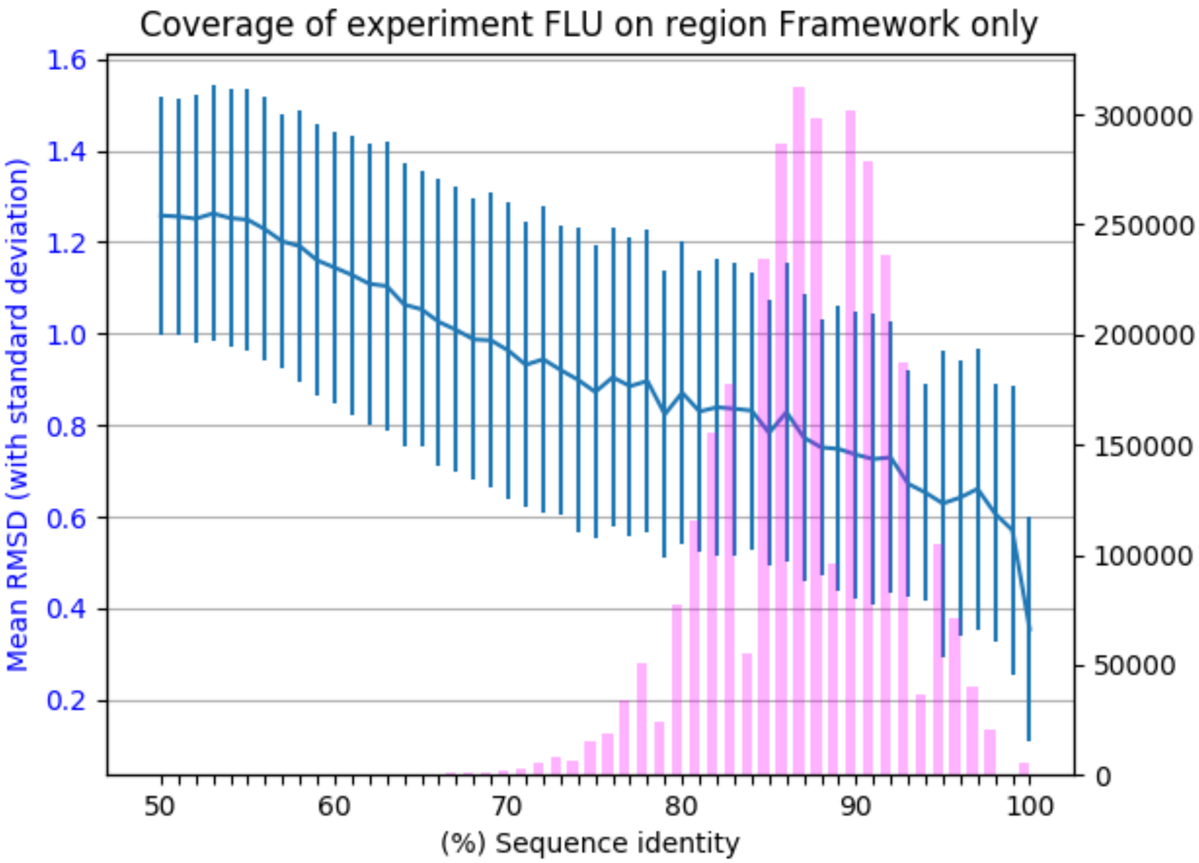

Supplementary Figure 16. Structural coverage of framework sequences in FLU dataset. The pink bars indicate the number of sequences (right-hand y-axis) whose highest sequence identity structure match has the sequen**ce** identity given on the x-axis. The blue line (left-hand y-axis) indicates the expected Root Mean Square Deviation (RMSD) of a model built using a sequence identity match of that quality (with vertical standard deviation error bars).

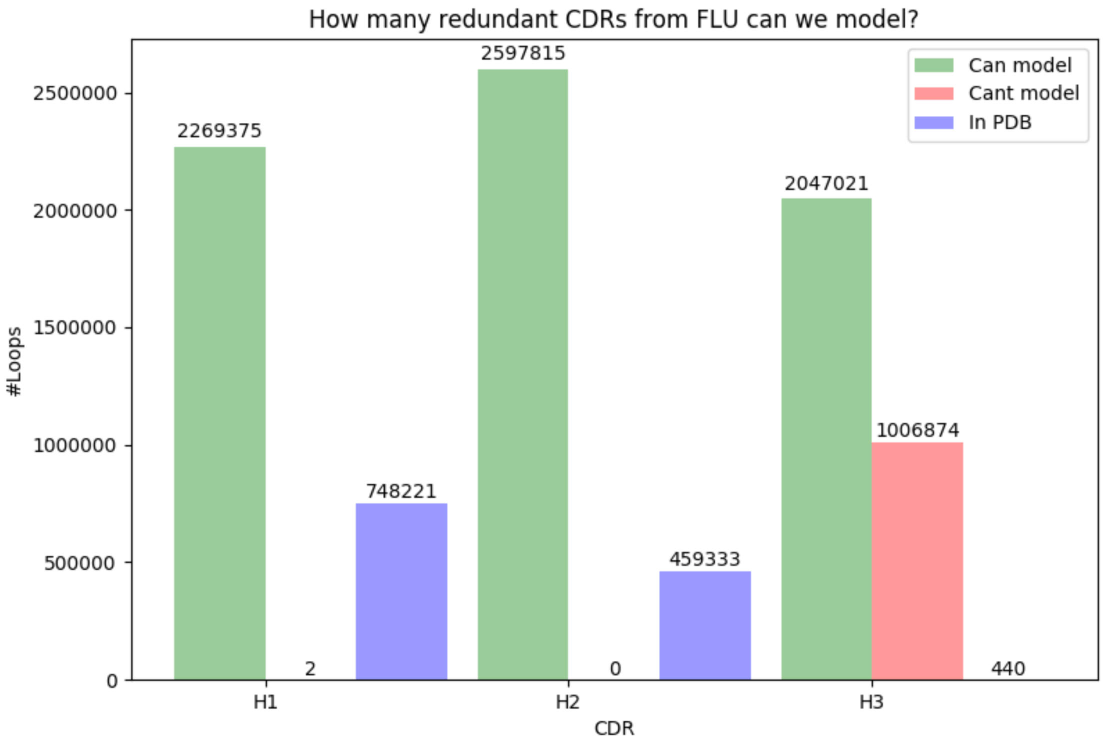

Supplementary Figure 17. Structural coverage of redundant CDRs in FLU. Blue bars indicate CDRs we found direct sequence matches in the PDB for. If no direct PDB match was found but we could produce a structural model, we indicate using green bars. The loops for which we could not find direct matches in the PDB and we could model are indicate by red bars.

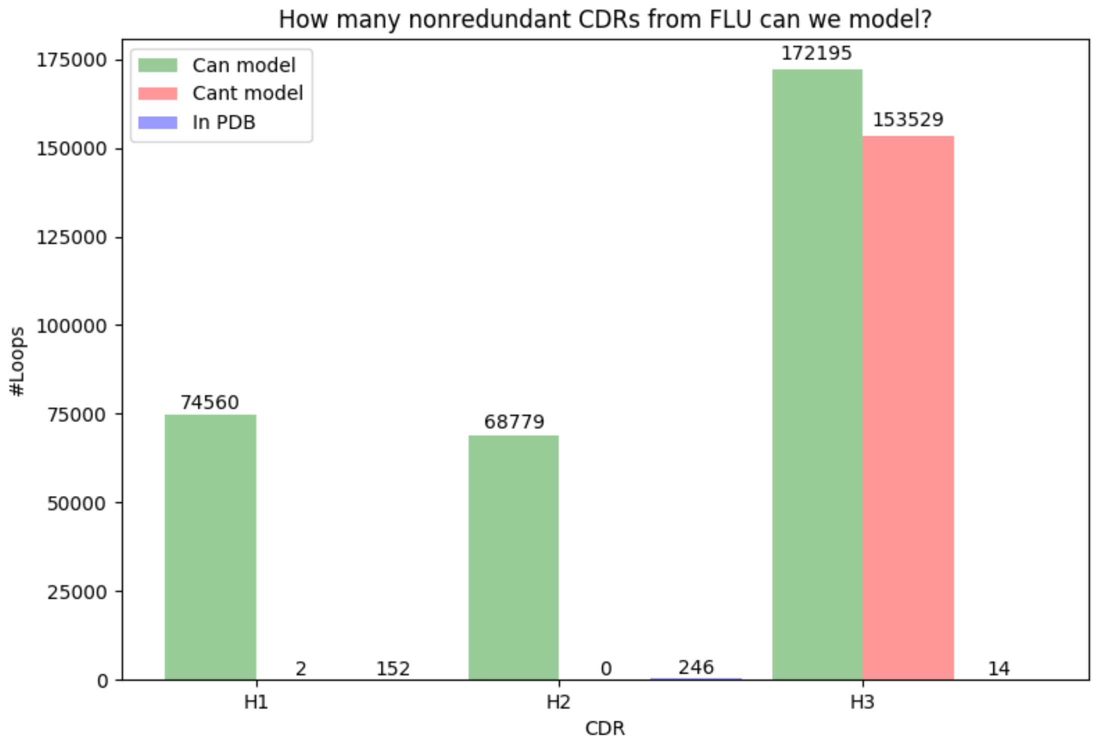

Supplementary Figure 18. Structural coverage of nonredundant CDRs in FLU. Blue bars indicate CDRs we found direct sequence matches in the PDB for. If no direct PDB match was found but we could produce a structural model, we indicate using green bars. The loops for which we could not find direct matches in the PDB and we could model are indicate by red bars.

Section 3. Direct heavy chain CDR matches to the PDB.

In Tables 1 and 2 we present the most direct matches to the PDB for CDR-H1 and CDR-H2 respectively. We indicate the redundancy in each of our five datasets as well as an indication whether the sequence is germline or not.

Table 1. H1 sequences for which we found direct matches in the PDB.

Sequence	FLU	HBB	HBP	MEN	UCBH	Germline
ASGFTFSSY	-	-	-	1	7	-
DYSITSGY	14	2	8	-	21	-
DYTFTDY	6	-	81	-	-	-
EYSFPNY	118	6	29	-	4	-
FTFSDY	61	-	104	42	-	-
FTFSNY	50	23	94	58	81	-
GASISDH	-	40	48	2	1	-
GASISDS	11	97	72	13	15	-
GASISSGGY	215	235	1487	100	445	-
GASVNNY	-	5	11	1	3	-
GDSIRSD	-	7	6	11	29	-
GDSITSA	-	9	3	-	-	-
GDSITSD	-	4	17	2	57	-
GDSMNNY	-	73	247	-	61	-
GDSVSSNSA	11885	50802	50090	86702	264506	Germline
GDSVSSYNA	1	551	224	33	181	-
GDSVTSD	-	2	-	1	21	-
GDSVTSG	-	-	-	1	1	-
GDTFIRY	11	10	13	-	7	-
GDTFSSY	5679	1910	4359	587	1378	-
GDTFTSY	1532	1070	648	386	754	-
GDTVSSNRA	-	-	-	-	154	-
GFDFDNA	-	-	2	-	-	-
GFDFSTY	66	211	532	75	110	-
GFIFSDN	12	25	313	89	31	-
GFIFSNY	-	-	16947	-	-	-
GFKFGDH	-	13	9	-	-	-
GFNISSY	-	-	-	-	9	-
GFNLSSS	-	-	11	1	4	-
GFNVSSS	-	258	13	3	7	-
GFNVSYS	-	9	196	-	-	-

GFSFDEY	478	224	377	40	-	-
GFSFSDF	-	448	-	-	640	-
GFSFSSH	-	1377	3840	-	1510	-
GFSFSSY	10546	14647	38642	19526	18712	-
GFSITSDY	-	2	1	-	96	-
GFSITSPY	-	-	1	-	-	-
GFSLIDY	3	-	-	-	-	-
GFSLIGY	-	2	-	-	-	-
GFSLNTY	7	28	159	5	60	-
GFSLRTSGM	1974	848	985	639	-	-
GFSLRTSRV	6	13	8	243	-	-
GFSLSDK	-	-	1	-	-	-
GFSLSGF	1	26	24	2	21	-
GFSLSSN	144	27	117	17	25	-
GFSLSSSAM	1	6	14	3	-	-
GFSLSSY	-	779	3417	-	1007	-
GFSLSTAGM	139	39	240	355	-	-
GFSLSTSGM	13832	23072	19249	22028	-	Germline
GFSLSTSNM	-	-	4	7	-	-
GFSLSTY	237	388	1376	576	274	-
GFSLSTYGM	426	158	199	147	-	-
GFSLTDY	147	117	102	-	75	-
GFSLTGA	-	-	-	-	5	-
GFSLTGY	2	3	27	-	26	-
GFSLTNY	-	193	501	26	106	-
GFSLTSY	157	128	82	112	66	-
GFSLTTSGI	6	8	41	2	-	-
GFSLTTY	4	233	92	29	61	-
GFTFDDG	-	4	2	-	1	-
GFTFEDY	2985	1097	5340	2264	688	-
GFTFGHY	1273	142	1005	284	288	-
GFTFGSF	622	783	1697	3179	539	-
GFTFIDN	1	2	3	3	10	-
GFTFNDY	2190	3102	8854	1516	1767	-
GFTFNNY	9835	11127	28473	35839	10770	-
GFTFNTD	-	24	-	-	36	-
GFTFRKF	-	-	453	-	-	-
GFTFRNY	6024	7715	24476	37814	6947	-
GFTFS	-	8	9	106	-	

GFTFSAY	6431	-	19685	-	6894	-
GFTFSDA	832	3083	4739	2894	5547	-
GFTFSDY	34840	93205	127924	93701	71179	Germline
GFTFSGY	10505	15611	29756	19989	16331	-
GFTFSHY	2983	3874	16058	4119	4783	-
GFTFSNF	5302	6675	16543	36595	7059	-
GFTFSNH	-	-	9344	4648	5143	-
GFTFSNT	-	621	735	-	1229	-
GFTFSNY	46429	79705	166333	153997	105136	-
GFTFSPY	870	1920	3277	-	-	-
GFTFSRA	-	-	1615	-	-	-
GFTFSRY	13877	19696	55460	27943	18259	-
GFTFSSF	-	12073	27790	22271	-	-
GFTFSSS	5164	8921	17705	36251	15331	Germline
GFTFSSY	118364	763879	532132	927002	542568	Germline
GFTFSTF	4410	-	6747	4688	5452	-
GFTFSTY	38992	56755	132309	133213	83352	-
GFTFSVY	1855	2179	3173	999	1198	-
GFTFTDF	1115	341	827	103	165	-
GFTFTDY	3209	3107	8440	3327	2893	-
GFTFTNY	5012	9178	15727	14915	10497	-
GFTFTST	-	27	22	42	41	-
GFTISDY	1062	1024	247	117	152	-
GFTISNS	11	18	92	-	40	-
GFTISNY	-	250	495	-	-	-
GFTISSN	422	-	533	875	-	-
GFTLDSY	-	-	-	-	13	-
GFTLDYY	-	-	-	3	-	-
GFTLRTY	-	-	1045	-	-	-
GFTLSGD	5	3	25	4	4	-
GFTLSGS	30	463	1159	263	231	-
GFTLTGL	-	-	7	-	-	-
GFTVSSN	6291	54834	39562	62567	30007	Germline
GGNFNTY	2	49	466	-	2	-
GGPFRSY	844	14	65	-	28	-
GGPINNA	-	-	-	2	-	-
GGPLRNY	305	7	2	-	-	-
GGS	-	-	-	-	-	
GGSFSSY	4055	2518	4680	2931	1142	-

GGSFSTY	1128	1011	3560	-	268	-
GGSIRRN	-	1	1	2	1	-
GGSIRSGGY	73	231	1166	168	221	-
GGSIS	-	-	1	-	-	-
GGSISGF	172	123	687	178	643	-
GGSISGGY	87	8	107	24	28	-
GGSISNN	105	47	306	10	366	-
GGSISNY	3385	2002	6935	3063	8848	-
GGSISS	28	5	40	170	8	-
GGSISSSY	17	173	1003	392	492	-
GGSISSY	13511	45227	40563	43429	68958	Germline
GGSISTN	189	232	199	38	98	-
GGSLSSF	64	17	215	8	53	-
GGSMINY	1	5	24	2	89	-
GGTFDSY	131	89	396	9	8	-
GGTFN	249	-	-	-	-	-
GGTFNRL	-	10	-	-	-	-
GGTFNSY	3853	1867	6236	1087	578	-
GGTFRTY	1638	318	650	595	395	-
GGTFS	5	-	-	-	30	-
GGTFSMY	22	29	23	2	9	-
GGTFSNY	11344	7352	15907	5920	5520	-
GGTFSSY	37006	203509	64177	111709	144341	Germline
GGTFSTY	10747	4578	11840	2649	2590	-
GGTLRTY	11	59	87	11	1	-
GGTLSNY	492	292	526	35	118	-
GGTSNNY	32	26	71	273	2	-
GISLSRY	-	-	-	-	1	-
GITFSGY	-	-	106	-	-	-
GITFSRY	-	150	1485	625	-	-
GLSLTSY	1	-	1	2	1	-
GLTFSAN	-	-	-	2	-	-
GLTFSNY	1037	3089	4651	3340	2936	-
GRAFSSY	5	-	-	-	-	-
GRSFSNS	-	-	-	-	1	-
GRTFRNY	-	-	2	-	-	-
GRTFSDY	-	3	-	-	-	-
GRTFSSD	1	-	1	1	1	-
GRTFSSF	1	3	7	4	-	-

GRTFSSY	467	96	56	46	169	-
GRTFSTY	9	-	14	-	-	-
GRTLSSY	1	2	-	-	4	-
GSIFSIY	4	-	-	-	-	-
GSISSIN	-	-	3	-	-	-
GSSFTGY	-	-	-	-	27	-
GSTLNNY	16	3	9	-	8	-
GTSFDDY	-	-	2	-	-	-
GVSFSSF	4	5	256	-	16	-
GVTFSNV	-	57	6	3	14	-
GYAFSSS	2	2	2	1	45	-
GYDFTHY	-	-	6	4	8	-
GYGFINY	16	-	13	19	-	-
GYIFTDF	512	842	-	-	270	-
GYISTDH	1	-	-	1	-	-
GYKFSSY	34	502	313	23081	46	-
GYKFTDY	366	156	359	45	76	-
GYNFIDY	520	460	569	81	118	-
GYNFNDY	-	109	-	6	398	-
GYNFREY	-	-	53	-	-	-
GYNFTSY	1523	1376	2380	1140	1365	-
GYRFTSY	4643	3405	6077	4481	3973	-
GYSFIDY	-	385	708	-	-	-
GYSFNFY	1	-	-	-	-	-
GYSFSDY	1958	891	3042	605	989	-
GYSFSNY	3971	3577	8148	5035	6410	-
GYSFSRY	202	175	467	-	-	-
GYSFTDY	-	-	5414	1813	-	-
GYSFTGH	35	252	224	201	248	-
GYSFTGY	4579	2617	6528	1536	4196	-
GYSFTNF	341	419	1736	1003	1420	-
GYSFTNY	13072	14667	25802	35209	36110	-
GYSFTSF	-	-	2587	-	2015	-
GYSFTSN	772	913	2151	3206	2074	-
GYSFTSY	21330	150674	53425	149403	163473	Germline
GYSFTTY	7607	9921	17291	29796	33773	Germline
GYSISSGY	7175	1511	3590	7058	27426	Germline
GYSISSNY	7	6	100	3	318	-
GYSITNNY	-	1	-	-	1	-

GYSITSDF	-	1	-	-	3	-
GYSITSDY	1	8	18	31	91	-
GYSITSGY	621	87	193	127	1674	-
GYSITSNY	29	-	-	-	86	-
GYSITSTY	-	-	-	-	6	-
GYSITTNY	-	1	1	-	11	-
GYSLSTSGM	14	12	13	8	-	-
GYTDSRY	-	1	-	-	-	-
GYTFINY	5836	5693	10363	2086	4245	-
GYTFNDY	959	866	1453	241	-	-
GYTFSDY	8086	-	9073	-	-	-
GYTFSSN	529	325	922	126	351	-
GYTFSSY	8153	10892	21724	21325	11938	-
GYTFSTS	-	142	398	-	280	-
GYTFT	5	-	4	-	18	-
GYTFTAY	10019	6568	-	-	5260	-
GYTFTDF	2505	-	-	-	-	-
GYTFTDH	1140	987	2616	813	939	-
GYTFTDN	-	-	351	-	-	-
GYTFTDY	22290	21346	31276	19803	18396	Germline
GYTFTEY	1758	995	865	65	350	-
GYTFTGN	2403	468	883	8706	567	-
GYTFTGY	27676	82856	43503	49800	61452	Germline
GYTFTHY	3995	-	1778	3944	-	-
GYTFTNY	24797	31597	64917	61757	50342	-
GYTFTSD	-	895	1005	308	1097	-
GYTFTSN	3376	2519	6576	8744	3205	-
GYTFTSS	-	-	2794	-	-	-
GYTFTSY	62463	366099	161077	337316	260331	Germline
GYTFTTY	20928	19565	46007	46165	39301	-
GYTLTEA	238	-	-	-	-	-
GYTLTEL	1811	41463	10185	20115	10819	Germline
GYTY	65	-	-	-	2	-
GYVFSSS	-	-	1	1	-	-
RGTLSSY	7	8	6	-	-	-
RNTFTDY	1	1	1	-	-	-
SYSITSDY	-	-	-	-	2	-

Table 2. H2 sequences for which we found direct matches in the PDB.

Sequence	FLU	HBB	HBP	MEN	UCBH	Germline
ASHGNV	-	-	1	-	-	-
AVSGN	-	3	-	-	-	-
AYDSSN	-	2	-	-	-	-
DNDGT	-	-	3	-	3	-
DPANGA	-	-	-	-	3	-
DPANGD	-	3	21	-	11	-
DPANGH	-	-	5	-	-	-
DPANGN	-	2	4	34	10	-
DPASGN	-	-	5	1	2	-
DPATGN	-	-	3	-	-	-
DPEDAD	235	9	10	3	-	-
DPEIGD	-	-	1	-	-	-
DPENGD	4	41	27	3	1	-
DPENGN	-	2	3	8	-	-
DPEQGN	1	-	1	-	-	-
DPESGE	8	308	394	108	2	-
DPETGG	8	2	4	-	-	-
DPNGGG	25	34	1798	11	32	-
DPNSDV	5	-	5	-	-	-
DPNSGG	1142	1271	1247	345	979	-
DPQDGE	192	163	147	46	7	-
DPSDGE	1	-	17	-	-	-
DPSDSE	401	348	1202	22	102	-
DPSDSY	9599	60470	38697	32831	25880	Germline
DPSGGR	16	25	161	17	442	-
DPSNGD	18	11	4	-	5	-
DPSNGR	4	5	2	-	2	-
DPSNSY	75	1535	361	121	465	-
DPYNGD	4	4	215	-	-	-
DSGGGG	5	13	3	-	5	-
DSSGR	208	7	12	175	40	-
DTGGG	-	2	6	3	94	-
DTGGS	-	18	37	43	26	-
DTGGT	2	6	6	2	21	-
DTGNGN	20	64	61	21	311	-
DTNTGN	78	350	458	476	148	-

DTSGT	-	31	57	-	9	-
DWDDD	33162	30536	31217	27823	-	Germline
FGSGGN	-	3	6	-	3	-
FHTGE	1	-	2	-	2	-
FPGDGD	30	-	4	-	6	-
FPGDGS	-	-	-	-	3	-
FSDGS	182	38	25	14	156	-
FYTGT	1846	442	2071	134	366	-
GFSGS	-	-	1	1	2	-
GNKANGYT	-	6	4	2	7	-
GPEENE	-	-	1	-	-	-
GPSGD	1	154	10	114	52	-
GSDGSI	1	1	4	3	4	-
GSRGTY	2	1	7	-	1	-
GSS	10	8	30	40	1	-
GTAGD	1243	8555	9361	9849	7826	Germline
GTDNGN	2	1	3	17	4	-
GTRGGR	-	-	2	-	-	-
GTSGN	1	23	41	2	13	-
HDSGD	-	-	74	-	74	-
HHSGD	211	266	651	7	197	-
HKSGD	-	-	-	-	2	-
HPHNGA	-	-	-	-	1	-
HPNSGA	12	307	118	36	145	-
HPNSGD	14	71	306	31	133	-
HPNSGN	113	86	613	102	104	-
HPNSGS	3	4	13	69	14	-
HPSDSE	2	124	25	41	259	-
HWRGT	-	-	10	-	-	-
HYRGT	129	51	376	5	130	-
HYSAG	-	5	23	-	13	-
HYSGN	1807	579	3177	384	1433	-
HYSGS	5885	3322	16031	6796	10617	-
IPAFGT	448	307	869	297	17	-
IPDFRT	-	-	9	-	-	-
IPFFGT	960	1147	2763	571	280	-
IPIFGI	1300	710	1174	3803	4575	-
IPIFGT	27599	136644	49551	91167	88859	Germline
IPIVDI	114	374	136	14	17	-

IPKYGT	93	6	14	1	-	-
IPLFGF	-	-	1	-	-	-
IPLFGK	118	44	454	26	38	-
IPLLTI	-	6	1	-	1	-
IPSNGG	1	1	-	-	7	-
IPTFRT	19	6	196	35	19	-
IPVLGT	1285	254	1035	591	97	-
IPWFGT	-	-	24	-	1	-
IYGGT	-	-	4	-	1	-
KDGS	1	-	-	-	-	-
KPRGGA	-	17	-	-	-	-
KQDGND	7	162	33	30	106	-
KSKTDGGT	5421	55679	23934	42846	30703	Germline
KSRTDGGT	276	788	1520	351	626	-
KYDGRN	-	1	19	-	-	-
LGGS	-	-	1	-	1	-
LPGSGD	-	-	-	-	1	-
LRDGT	-	3	2	2	1	-
LWNDG	-	1	-	-	-	-
MPIFDI	-	7	1	-	-	-
NAGDGS	167	116	48	19	433	-
NAGGGD	5	6	8	363	32	-
NGNSGY	-	4	4	-	1	-
NHSGS	7889	41026	34466	28373	10081	Germline
NLNGGR	-	-	2	-	1	-
NNEGTT	139	1	3	1	6	-
NNGGGR	-	17	-	-	2	-
NPANGN	8	125	520	5791	307	-
NPDGIT	-	8	-	-	2	-
NPDSST	-	-	-	-	10	-
NPDTDS	-	3	-	-	-	-
NPGNGN	1227	542	2777	991	3123	-
NPGNGY	4	26	136	63	68	-
NPGSDY	-	-	2	3	16	-
NPGSGD	168	40	92	79	309	-
NPHSGD	195	173	486	92	428	-
NPKSGD	2880	1274	2309	401	1005	-
NPKTGG	1454	609	2122	459	179	-
NPNNGG	1948	2017	2938	2047	1636	-

NPNNGR	23	18	36	3	15	-
NPNSGG	23686	82567	38856	45773	57175	Germline
NPNSGY	114	345	1067	247	174	-
NPNTGY	5	21	93	8	17	-
NPQSGG	348	52	282	71	20	-
NPRGGG	27	196	404	30	36	-
NPRNGD	11	22	274	9	28	-
NPRNGG	1021	12	62	7	20	-
NPRTGD	159	29	79	-	3	-
NPSGGS	13364	84211	37284	58357	65794	Germline
NPSNGG	365	67	177	10	132	-
NPSRGY	1	-	2	-	-	-
NPSSGY	7	36	166	72	27	-
NPTSGG	515	177	740	400	262	-
NPYKGV	29	-	-	-	-	-
NPYNDD	-	5	18	-	2	-
NPYNDG	1	-	4	4	-	-
NPYNGG	670	59	2320	12	12	-
NPYNGN	844	440	2803	197	477	-
NPYNSG	3	-	8	-	1	-
NPYTGE	-	-	-	4	19	-
NPYYGS	-	8	1	-	-	-
NRSGS	159	275	1101	168	48	-
NSDGG	-	10	19	5	19	-
NSDGST	2132	3138	4570	7496	2610	-
NSDSTY	-	5	350	-	5	-
NSNGAS	-	9	8	-	17	-
NSNGDK	-	13	-	25	-	-
NSNGGN	1	26	60	5	45	-
NSVGD	-	4	-	-	3	-
NSVGSS	9	74	33	39	213	-
NTETDE	-	3	-	-	-	-
NTETGD	-	9	7	-	11	-
NTHTGE	-	3	2	-	5	-
NTNGGF	-	-	3	-	-	-
NTNTGE	97	37	166	33	14	-
NTQSGV	-	-	-	-	2	-
NTRGGI	-	-	2	-	-	-
NTRSGV	-	6	1	-	-	-

NTYSGV	2	-	2	-	-	-
NTYTGE	3	7	36	1	28	-
NTYTGR	313	1	561	-	5	-
NWDSAR	-	-	38	-	-	-
NWNGGD	1	29	462	6	25	-
NWSGTT	-	-	5	-	1	-
NWSSGR	1	6	5	1	-	-
NYDGSS	62	313	388	58	52	-
NYSGF	13	-	141	199	2	-
NYSGT	335	307	1730	122	902	-
NYSGY	-	14	121	292	39	-
RDKAKGYT	-	-	-	44	2	-
RNKAKGYT	1	20	38	7	67	-
RNKANGYT	30	377	262	195	584	-
RNKANNHA	-	2	2	1	22	-
RNKGNGYT	-	5	41	-	13	-
RNKPKGYT	2	7	2	-	19	-
RNKRNGDT	-	-	4	-	-	-
RNKVNGYT	13	13	41	12	140	-
RPYSGE	-	1	-	-	-	-
RSGGGR	-	2	-	-	-	-
RSKSNNYA	1	226	186	111	43	-
RSKVNNHA	-	3	6	-	1	-
RSSDGT	-	41	2	41	9	-
RTKPNNYA	191	47	68	72	62	-
RYGGG	-	2	1	-	3	-
SAGGDK	1	17	12	-	32	-
SAGGGS	328	671	1279	620	486	-
SAGTGN	4	7	32	8	19	-
SAHGGS	-	2	5	-	3	-
SASGGS	3349	2511	3315	5166	2344	-
SASGS	8	78	380	21	384	-
SDDGSL	-	5	83	1	27	-
SDGGAY	-	1	-	-	-	-
SDGGSF	-	2	-	-	-	-
SDGGSY	-	4	5	-	-	-
SDGGTY	1	1	29	7	-	-
SDRES	-	-	5	-	1	-
SDSDGR	-	4	14	1245	11	-

SEGGI	-	-	1	-	-	-
SFSGN	86	14	304	4	47	-
SFSGS	30	124	409	320	77	-
SGDGRL	1	1	128	-	3	-
SGGGH	-	1	119	2	2	-
SGGGRN	3	10	124	20	10	-
SGGGS	154	136	336	218	193	-
SGGGTY	2	47	354	1	16	-
SGNSLY	11	3	5	92	-	-
SGSGGN	2729	5578	8655	6249	4929	-
SGSGGS	14028	167828	80013	188246	91398	Germline
SGSSGD	7	52	122	187	140	-
SGSSRY	27	106	170	103	46	-
SGYSGD	222	810	611	58	43	-
SISGGS	32	89	405	155	141	-
SKGGGS	-	12	1	-	1	-
SNGGGS	9	100	341	147	49	-
SNGGGY	-	6	-	3	2	-
SNGGSY	2	12	15	-	5	-
SNLDGS	-	-	2	-	-	-
SNSGGN	138	101	114	55	398	-
SPAGGY	-	-	3	-	-	-
SPDGGS	-	30	74	1	72	-
SPGGSN	-	11	6	36	3	-
SPGNGD	8	4	31	1	52	-
SPGSSS	-	1	2	-	2	-
SPIFGS	3290	7	20	81	-	-
SPSTGR	1	1	1	-	1	-
SPYSGD	148	287	468	423	108	-
SPYSGS	5	187	14	17	2	-
SPYSGV	1	1	2	-	6	-
SPYSSS	-	-	-	2	1	-
SPYYGS	-	9	6	-	-	-
SQGGDI	-	2	-	-	-	-
SQSGAA	-	-	-	-	7	-
SRGGGY	-	-	4	1	2	-
SRGGS	18	48	64	5	52	-
SRGGSY	3	4	84	-	-	-
SRNGAN	10	2	1	7	4	-

SRSGGL	4	-	55	33	-	-
SRSGGS	15	243	485	214	327	-
SRSGSV	-	10	12	-	54	-
SRSGSY	6	51	77	55	10	-
SRSGTL	-	-	2	-	3	-
SRSSGD	-	2	37	-	2	-
SRTSKT	1	1	1	-	-	-
SRYN	-	1	-	-	-	-
SSAGDR	-	-	13	1	-	-
SSDGRT	143	505	472	120	129	-
SSDGTD	130	143	68	22	57	-
SSDSSN	3	8	15	2	28	-
SSFGS	-	4	-	-	8	-
SSGGAY	164	43	26	-	21	-
SSGGG	9	40	78	55	70	-
SSGGGN	6	144	150	44	107	-
SSGGGR	-	16	59	11	14	-
SSGGGT	376	865	562	711	807	-
SSGGI	-	17	288	26	25	-
SSGGR	3	110	500	22	44	-
SSGGS	1251	325	937	280	636	-
SSGGSS	24	187	218	2331	414	-
SSGGSY	2	413	583	177	143	-
SSGSST	196	1005	2888	4987	678	-
SSKGGS	3	41	138	10	21	-
SSPGT	3	-	-	-	-	-
SSRDGS	73	1	36	58	7	-
SSSDGS	8	37	32	23	84	-
SSSGDP	-	3	65	24	38	-
SSSGGM	-	194	165	14	10	-
SSSGGS	1377	2586	3419	2860	3110	-
SSSSGY	383	1103	1402	912	972	-
SSSSSY	4011	127900	43330	90710	45926	Germline
SSSYGS	5	1	41	-	-	-
SSSYGY	1	1	-	-	-	-
SSTDGT	-	1	6	2	4	-
SSTGRT	10	118	227	367	79	-
SSTGTS	-	12	146	2	22	-
SSYSGY	1	5	4	-	-	-

STGDN	-	-	2	1	-	-
STGGYN	-	-	-	-	1	-
STSSTT	2	135	323	100	93	-
STSSTY	247	993	1159	1259	330	-
STYSGD	145	381	1254	23	42	-
STYSGY	10	22	22	8	1	-
SVYSGN	37	180	601	120	86	-
SWNSGS	10661	31285	22833	43312	22361	Germline
SWNSNI	-	32	20	-	9	-
SWSGGS	6	33	272	33	27	-
SWSGGT	6	23	24	11	18	-
SWSSGT	30	54	105	18	31	-
SWTGAN	-	-	-	-	1	-
SWTGGL	-	-	-	-	2	-
SYDGEN	8	58	118	7	12	-
SYDGRH	16	122	663	37	32	-
SYDGRN	691	2793	7982	1389	1994	-
SYDGS	26	240	715	396	152	-
SYDGSK	1790	5047	14773	3802	2886	-
SYDGSN	12150	144939	86997	116511	82965	Germline
SYDGST	842	973	5404	756	1622	-
SYGGL	-	2	-	-	1	-
SYNGSS	3	24	17	6	-	-
SYRGS	20	265	891	187	480	-
SYSAN	-	8	67	-	1	-
SYSGD	70	263	564	72	150	-
SYSGF	2422	36	264	324	23	-
SYSGI	121	125	892	288	646	-
SYSGN	958	664	3439	1397	1721	-
SYSGR	940	270	2013	5021	446	-
SYSGS	5214	3107	15402	6471	7612	-
SYSGSE	-	-	1	-	1	-
SYSGT	738	809	4721	443	1733	-
TGEGDS	-	-	17	-	-	-
TGSGRS	4	38	102	20	22	-
TISGY	-	-	1	-	-	-
TNGGS	-	24	11	1	20	-
TNSGGR	-	29	388	27	15	-
TNTGV	-	1	-	-	-	-

TPILGI	33	255	102	39	77	-
TPLLGT	3	11	58	38	1	-
TPYNGA	-	1	-	-	49	-
TREGS	-	-	1	-	1	-
TRSGG	2	1	230	-	95	-
TSGDT	48	6	159	-	6	-
TSGGS	141	64	553	263	125	-
TSHGDR	-	1	2	-	-	-
TSNGDN	-	30	225	63	27	-
TSSGDK	-	12	21	-	91	-
TSSSIN	22	3	-	-	1	-
TTTGT	1	-	45	1	16	-
TYDGS	1	10	88	32	1	-
TYSGS	59	126	763	31	138	-
TYSGT	127	13	203	1	56	-
TYSGTT	-	2	2	57	1	-
VPYSGG	-	-	-	-	3	-
VSGGN	-	-	-	-	1	-
VSSGGS	-	-	40	-	16	-
WAGGS	-	3	-	-	-	-
WAGGT	-	-	-	1	-	-
WDDGSD	3	14	29	15	79	-
WFDENN	1	9	11	-	25	-
WGDGN	-	-	1	1	4	-
WGDGR	-	-	-	-	1	-
WGDGS	1	-	1	-	1	-
WGGGT	7	-	-	-	-	-
WSGGG	-	-	3	-	1	-
WSGGN	-	-	3	-	2	-
WSGGS	-	17	4	3	12	-
WTGGS	1	-	6	-	-	-
WWDDD	311	2	8	-	-	-
WWDDV	1	-	-	-	-	-
WWDGD	1	-	-	11	-	-
WWNDD	1	-	4	-	-	-
WYDGDN	5	94	179	54	80	-
WYDGSN	12358	82961	69645	78889	50613	Germline
WYNGSR	3	1	5	-	-	-
WYSGSN	625	-	6	-	5	-

YAGSGG	-	-	-	-	1	-
YDSGD	15	14	36	15	168	-
YGSGGS	2	11	244	15	53	-
YGSSGR	-	-	-	-	1	-
YHSGN	2113	907	7376	811	12108	-
YHSGS	12477	16531	43352	30329	51366	Germline
YHTGV	10	14	164	3	154	-
YPADSD	5726	3786	6283	8318	6721	-
YPDDSD	8349	5642	14546	5343	11037	-
YPGDFD	82	604	8536	723	1548	-
YPGDGD	70	18	93	10	46	-
YPGDSD	56932	147912	105037	193685	216539	Germline
YPGDSE	5072	5328	11568	9469	10019	-
YPGDSY	640	314	815	206	1493	-
YPGGGS	5	-	-	-	-	-
YPGNGD	2	15	54	1	14	-
YPGNGH	-	-	22	-	2	-
YPGNGN	1	9	104	77	16	-
YPGNSA	2	20	29	4	38	-
YPGNSD	1749	967	1814	933	3107	Germline
YPGS	-	-	-	1	-	-
YPGSGG	2	4	1	-	-	-
YPGSGN	1	-	5	-	3	-
YPGSGS	-	3	2	-	5	-
YPGSSY	-	-	-	-	2	-
YPNNGG	53	11	5	26	58	-
YPNNGV	-	-	1	-	-	-
YPRSGN	1	2	-	1	-	-
YPSDSY	8	100	90	27	482	-
YPYNGG	1	-	2	-	-	-
YPYSGY	-	6	-	-	-	-
YSDDD	63	49	325	53	1	-
YSGGS	6396	55603	50908	67765	26000	Germline
YSSGS	6520	3678	14154	6073	7245	-
YSSSGS	-	-	-	4	-	-
YTDGN	3	56	63	8	74	-
YTHTGN	-	4	-	-	-	-
YWDDD	49548	57141	91907	105274	4	-
YYRSGWY	22	238	1399	44	325	-

YYRSKWF	1504	1932	6184	3167	25832	-
YYRSKWY	15077	64028	53611	116533	396012	Germline
YYSGS	48118	108264	158066	113821	143301	Germline

Supplementary Materials and Methods

Sequencing Techniques.

<u>Commercially sourced RNA samples.</u> Three different total RNA samples at 1µg/µl were sourced from Clontech Laboratories comprising 50µg prepared from normal human spleen pooled from 12 male/female Caucasians aged between 18 and 54 years (SP), 10µg from normal bone marrow from 56 Asian males aged between 22 and 85 (BM) and 10µg from normal human peripheral leukocytes from 426 males/females Asians aged between 18 and 54 (PBL).

<u>Reverse transcription and C-region specific oligonucleotides.</u> Multiple reverse transcription (RT) reactions were done for each RNA sample (SP: 12xVH,6xVK,6xVL BM: 6xVH, 2xVK,2xVL PBL: 6xVH,2xVK,2xVL) using human antibody constant region reverse oligonucleotides specific for the 3' ends of the CH1 gene of IgM and the 3' ends of the C-kappa and C-lambda genes. After an initial denaturation of the RNA at 65°C for 5 min in the presence of oligonucleotide and dNTPs multiple 20µl reactions each containing 1ug RNA, 200U Superscript III (Life Technologies), 20U RNasin (Promega), 5mM DTT, 500µM dNTPs and 1µM oligonucleotide were incubated for 60 min at 50°C and 15 min at 70°C before being frozen at -20°C.

<u>Primary PCRs.</u> Twelve family-restricted primary PCRs (5xVH, 4xVK, 3xVL families) were done on each of the three cDNA template samples SP, BM and PBL. Where a V-gene family required more than one oligonucleotide these were mixed in proportions equivalent to estimates of sequence frequency from previous human IgG RACE data (unpublished). A total of 304 individual (192xVH, 64xVK and 48xVL) 25µl buffered reactions, each with 1µl of cDNA as template, consisted of 1mM dNTPs, 1.5mM $MgSO_4$, 4µM forward and 4µM reverse oligonucleotides and 0.5U KOD hot start DNA polymerase (Merck Millipore). After an initial denaturation step of 96°C for 2 min, PCR cycling conditions for all reactions were 96°C for 15s, 55°C for 15s, 72°C for 15s for 40 cycles followed by a final extension step for 5 min at 72°C.

<u>Secondary PCRs.</u> An equivalent 304 (192xVH, 64xVK, 48xVL) 50µl individual secondary PCRs were done, keeping the DNA samples from the primary reactions separate in order to maximize V-gene diversity. The reactions, each with 2µl of primary PCR as template, had matched components and cycling conditions to the primary reactions except the cycle number was reduced to 30. Once again V-region family oligonucleotide sets were kept separate and members within each family were mixed at the pre-determined proportions.

<u>Sample preparation for Oxford sequencing centre.</u> The secondary PCR products for each of the specific V-gene family (VH1-6, VK1-4, VL1-2) were pooled, giving 12 samples. Approximately 1µg from each pool was analysed by agarose gel electrophoresis (Invitrogen UltraPure™ agarose) and the DNA of approximately 400bp was excised, gel extracted (Qiagen) and eluted into 50µl of water at a final concentrations of between 10-75ng/µl to be analysed by paired-end next generation

sequencing on an Illumina MiSeq machine at the Oxford Genomics Centre (OGC) at the Wellcome Trust Centre for Human Genetics, Oxford.

Bioinformatic Annotation of sequences.

Generation of the sequence files and gene annotation. IgBLAST 1.4.0 was used with Human V, D & J germline reference sets from IMGT for both heavy and light chains to germline annotate the full length reads. A custom Java pipeline was used to process the IgBLAST output and identify high quality sequences. The criteria for this were as follows: identified germline V & J genes; full length variable chain sequence (1-2 bp missing at 5' & 3' end was permitted); absence of stop codons or ambiguous nucleotide calls. Sequences successfully extracted were saved into flat files together with the identifiers of their assigned germline sequences.
